# Fludarabine Modulates Immune Response and Extends *In Vivo* Survival of Adoptively Transferred CD8 T Cells in Patients with Metastatic Melanoma

**DOI:** 10.1371/journal.pone.0004749

**Published:** 2009-03-09

**Authors:** Herschel Wallen, John A. Thompson, J. Zachary Reilly, Rebecca M. Rodmyre, Jianhong Cao, Cassian Yee

**Affiliations:** Department of Clinical Research, Fred Hutchison Cancer Research Center, Seattle, Washington, United States of America; Texas Children's Cancer Center, United States of America

## Abstract

**Background:**

Adoptive T cell therapy involving the use of *ex vivo* generated antigen-specific cytotoxic T lymphocytes provides a promising approach to immunotherapy. It has become increasingly apparent that anti-tumor efficacy using adoptively transferred T cells is linked to their duration of *in vivo* persistence and can only be achieved when combined with some form of pre-infusion patient conditioning regimen. An optimal conditioning regimen that provides a positive benefit without serious toxicities has yet to be defined. We have established a unique clinical model that allows for evaluation of a given conditioning regimen on adoptively transferred T cells in humans. In this first-in-human study (FHCRC #1796), we evaluate the use of fludarabine, an FDA-approved reagent with predictable lymphodepleting kinetics and duration of action, as a conditioning regimen that promotes homeostatic upregulation of cytokines and growth signals contributing to *in vivo* T cell persistence.

**Methods/Findings:**

We conducted a phase I study in patients with refractory metastatic melanoma. Patients received two infusions of a single tumor-reactive antigen-specific CTL clone expanded to 10^10^/m^2^; the first infusion was given without fludarabine conditioning, and the second CTL infusion was given after a course of fludarabine (25 mg/m^2^/day×5 days). This design permits intra-patient comparison of *in vivo* T cell persistence pre- and post-fludarabine. Nineteen CTL infusions were administered to ten patients. No serious toxicities were observed. Three of nine evaluable patients experienced minor response or stable disease for periods of 5.8–11.0 months with two additional patients demonstrating delayed disease stabilization. The median overall survival in this heavily pre-treated population was 9.7 months. Fludarabine led to a 2.9 fold improvement in the *in vivo* persistence of transferred CTL clones from a median of 4.5 days (range 0–38+) to 13.0 days (range 2–63+) (p<0.05). Fludarabine lymphodepletion increased plasma levels of the homeostatic cytokines IL-7 and IL-15. Surprisingly, fludarabine also increased the relative percentage of CD4+ T cells expressing the regulatory protein Foxp3.

**Conclusions/Significance:**

Lymphodepletion with fludarabine enhances transferred T cell persistence but suggest that additional improvements to optimize T cell survival and address regulatory T cells are critical in providing anti-tumor efficacy.

**Trial Registration:**

ClinicalTrials.gov NCT00317759

## Introduction

Adoptive T cell therapy involves the *ex vivo* isolation and expansion of tumor-reactive T lymphocytes for patient infusion and has proven to be a promising approach to cancer immunotherapy. Tumor-reactive effector cells of desired specificity and phenotype can be identified *in vitro*, selected based on expression of a high affinity receptor and specific surface markers and expanded to several billion in number. The use of a well-defined, uniform population of T cell clones facilitates *in vivo* tracking and provides a means of rigorously evaluating the influence of extrinsic immunomodulatory factors on the anti-tumor T cell response.

In phase I clinical trials, transfer of antigen specific CD8+ T (CTL) cells for the treatment of patients with metastatic melanoma led to encouraging clinical responses[1], [2]. Results using CD8+ (CTL) clones targeting MART-1 and gp100 in conjunction with low doses of IL-2 demonstrated that infused clones can be detected in appreciable numbers, traffic to tumor sites, and result in tumor regression[1]. However, in these studies, adoptively transferred CTL experienced a limited period of *in vivo* persistence and the duration of *in vivo* persistence has been closely linked to the clinical response[3].

In murine models, pre-infusion conditioning (in the form of chemotherapy or radiation-induced lymphodepletion) may extend the survival and function of transferred T cell *in vivo* through a variety of mechanisms including recruitment of homeostatic proliferative cytokines such as IL-7 and IL-15[4], [5], depletion of inhibitory regulatory T cells[6], [7], or potentially the creation of immunologic “space” . Prior host lymphodepletion has been shown in murine models to be important for the anti-tumor efficacy of adoptive cellular therapy[8], [9].

In a recent study reported by investigators at NIH[10], adoptive transfer of autologous tumor infiltrating lymphocytes following immunodepletion with high-dose cyclophosphamide and fludarabine resulted in tumor regression accompanied by *in vivo* T cell expansion and in some cases, virtual replacement of the entire peripheral T cell repertoire with the infused clone. It is not clear, however, that such a skewed reconstitution of the immune system is required for optimizing T cell persistence nor that such a profound level of immuno-depletion which was accompanied by serious and potentially life-threatening toxicities, is essential. We postulated that induction of a relative lymphopenia without attendant serious toxicities may be sufficient to upregulate *in vivo* homeostatic cytokines to the benefit of transferred T cells.

Fludarabine is a purine analog which induces lymphopenia when administered in a standard five day course. It reduces the reduces the average CD4 counts by ∼80%, with sustained lymphodepletion for approximately 4 weeks. While some risk associated with lymphopenia is observed in patients receiving multiple cycles of fludarabine therapy, the frequency and severity of infectious complications is significantly less and the period of lymphopenia is well-defined in patients receiving a single five-day course of fludarabine[11]. While fludarabine has significant effects on hematologic malignancies, it has no anti-tumor effect on solid tumors such as melanoma[12].

To evaluate the influence of fludarabine lymphodepletion, we established a unique clinical model that allows for rigorous evaluation of the influence of a given conditioning regimen on T cell therapy in humans. A single tumor-reactive antigen-specific CTL clone expanded to 10^10^ cells/m^2^ is used for both a first infusion given to patients without conditioning, and then, for a second infusion, *following* patient conditioning. This strategy allows intra-patient comparisons and eliminates any confounding variability associated with disparate behavior among T cell clones in different recipients. In this study, we evaluate the use of fludarabine, a lymphodepleting agent with predictable kinetics and duration of action, as a conditioning regimen to enhance the *in vivo* persistence of transferred T cells. We report the results of a phase I study, Fred Hutchison Cancer Research Center (FHCRC) Protocol #1796, in ten patients with refractory, metastatic melanoma receiving autologous CD8+ T cell clones targeting a melanoma-associated antigen (MART-1, gp100, or tyrosinase), without and with prior fludarabine lymphodepletion.

## Materials and Methods

### Ethics Statement

This study was conducted according to the principles expressed in the Declaration of Helsinki. FHCRC protocol #1796 received prior approval by the institutional review board at the Fred Hutchison Cancer Research Center. All patients provided written informed consent for the collection of samples and subsequent analysis.

### Generation of antigen specific CD8+ T cell clones

The protocol for this trial and supporting CONSORT checklist are available as supporting information; see Checklist S1, IRB approval S1, Flow Diagram S1, and Protocol S1. Peripheral blood mononuclear cells (PBMCs) were obtained by leukopheresis and antigen-specific CD8+ T lymphocytes were generated using autologous dendritic cells pulsed with the HLA-A2 restricted peptide epitopes MART-1 (M27:AAGIGILTV), gp100(G154:KTWGQYWQV), or tyrosinase (T368: YMDGTMSQV (40 ug/ml first stimulation, 10 ug/ml subsequent stimulations). After three cycles of stimulation at weekly intervals, T cells were cloned by limiting dilution and expanded for *in vitro* testing. CTL clones demonstrating rapid in vitro growth and specific lysis of antigen-positive tumor targets in a Cr^51^ release assay were selected for expansion. Clones were expanded in a 14 day cycles using anti-CD3 antibody (OKT3) at 30 ng/ml, irradiated allogeneic PBMCs at 10^6^/ml, irradiated allogeneic lymphoblastoid cell lines (2×10^5^/ml), and serial IL-2 at 50 U/ml every 2–3 days as described previously[1]. All clones demonstrated Class I MHC restricted antigen-specific cytolytic activity, characterized as CD3+CD8+CD4−, and subsequently cryopreserved. Clones were thawed and lot released for intravenous infusion after demonstrating viability >90%, cytolytic ability against A2+ targets, and sterility testing.

### Adoptive T cell therapy

**Figure 1:**
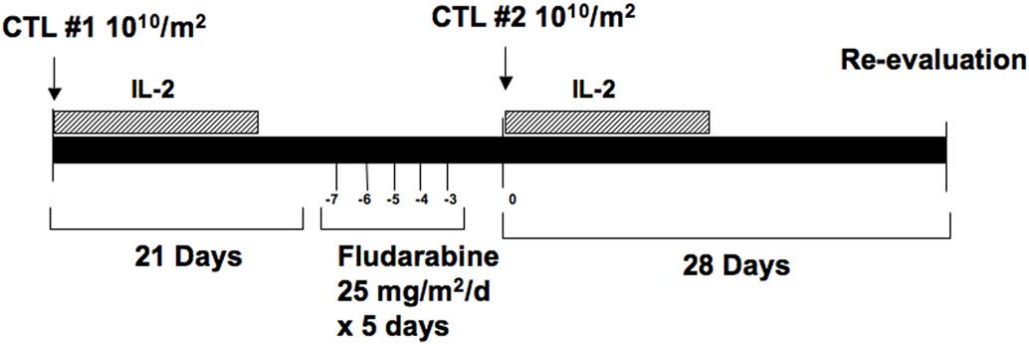
Treatment schema. Patients #1–4 received gp100, MART-1, or tyrosinase specific CD8+ T cell (CTL) infusions and fludarabine conditioning without IL-2. Patients #5–10 received very low dose IL-2 at 2.5×10^5^ s.c. bid for 14 days following each T cell infusion. Patients were evaluated for response four weeks after the CTL infusion #2 and every 2 months thereafter.

Ten patients were underwent treatment in a phase I study of adoptive T cell therapy. The procotol was open for recruitment from May 2003 through June 2006. All patients met the following entry criteria: histopathologic diagnosis of metastatic (stage IV) melanoma expressing antigens Mart-1, gp100, or tyrosinase by immunohistochemistry, HLA-A0201+, age<75, well-controlled CNS disease or absence of CNS disease, and no clinically significant cardiac, hepatic, renal, or pulmonary dysfunction. All patients had measurable disease refractory to standard or investigational treatments. 38 patients were initially screened and 17 patients met entry criteria for study (21 patients excluded were non-HLA02). Subsequently 6 patients were excluded for metastatic progressive disease in the brain. We were unable to generate T cells for one of the patients meeting entry criteria. Patients underwent leukapheresis before initiating other therapies in order that T cell clones could be generated *in vitro* and cryopreserved until needed. Antigen specific T cell infusions were administered at 10^10^ cells/m^2^ intravenously for both infusion#1 and #2 according to the study schema in **Fig. 1**. Two T cell infusions for each patient were planned. For a given individual patient, CTL Infusion #1 and #2 contained the same cryopreserved CTL clone. The initial four patients received T cell infusions without IL-2 supplementation. In the absence of any serious toxicity, the last six patients received very low-dose IL-2 at 2.5×10^5^ U/m^2^ twice-daily s.c. for 14 days following each T cell infusion as a source of helper support. The dose of IL-2 used is sufficient to saturate the high-affinity IL-2 receptor *in vivo*, and, when administered alone, has no anti-melanoma effect and minimal toxicity[13]. Twenty-one days after the first T cell infusion, patients received fludarabine conditioning at 25 mg/m^2^/day consecutively for five days. Two days following the last dose of fludarabine, a second infusion of T cells was administered. In previous studies[1], we have shown that transferred CTLs persist for less than 7 and 17 days, without and with IL-2, respectively; therefore by 21 days, sufficient time will have elapsed so that T cells from the first infusion could be adequately assessed for *in vivo* persistence before the second infusion. Clinical trial data was analyzed by HW and CY, and reviewed by all authors.

### Clinical Evaluation

Patients were monitored for toxicities based upon Common Toxicity Criteria v3.0[14]. Tumor measurements were obtained four weeks after the second T cell infusion and every two months thereafter. Clinical responses were evaluated according to RECIST criteria[15] with the additional criterion of Minor response, which is considered a decrease in the sum of target lesion diameters between 20–30%, insufficient to qualify for Partial response (30% decrease in the sum of the diameters). All patients were followed clinically and radiographically until death.

### Evaluation of *in vivo* persistence

Tetramers were generated according to the protocol of Altman[16] and described previously[1]. To evaluate the *in vivo* persistence of T cells in peripheral blood, PBMCs were prepared from samples drawn on day 0 (pre-infusion), and days 1, 3, 7, and weekly until at least day 28. Phlebotomy maybe delayed for one day due to logistical or technical reasons. After completion of the study, PBMC samples were thawed and analyzed simultaneously by staining with peptide-MHC tetramer-APC or PE (25–50 ug/ml), anti-CD8-PE-Cy7, anti-CD3PE or APC, and “dump” antibodies. Flow cytometric analyses were carried out on a minimum of 50,000 cells. The frequency of antigen-specific CTL is presented as the fraction of tetramer^+^CD8^+^ lymphocytes divided by the total number of CD8^+^ cells. The duration of persistence is defined as the duration of an increase in tetramer^+^ CTL frequency at least two fold greater than the frequency in the pre-T cell infusion sample. All patient samples were tested in duplicate. This method detects with high specificity a frequency of antigen-specific T cells as low as 0.1% CD8+ cells.

### Intracellular Foxp3 staining

The human antibodies APC and PE-conjugated anti-CD3, FITC and PE-Cy7 conjugated anti-CD8, FITC-conjugated anti-CD4, and FITC-conjugated anti-CD14/16/19 “dump antibodies,” were obtained from BD biosciences. PE-conjugated anti-Foxp3 (Ebiosciences) and intracellular staining was performed according to the manufacturer's instructions and modified as follows: 5×10^5^ cells were stained for surface markers for 30 minutes at 22°C, fixed for 30 minutes with 1× Fix/Perm buffer. After three washes, cells were permealized in Perm buffer with DNase I (100 U) for 30 minutes followed by three washes. Then cells were blocked with 2% fetal calf serum and stained with PE-conjugated anti-human foxp3 (Ebioscience, clone PCH101). The frequency of Foxp3+ CD4+ cells is presented as a fraction of the total number of CD4+ cells after gating on CD3+ cells.

### Plasma cytokine levels

Plasma samples were drawn prior to the first T cell infusion, immediately prior to the second T cell infusion (corresponding to 48 hours post-fludarabine), and timepoints 7, 14, and 21 days after the second T cell infusion. Plasma samples were frozen, thawed, and analyzed simultaneously by the Cytokine Analysis Facility at the FHCRC for IL-7, IL-15, and in selected cases IL-2 by sandwich ELISA. This assay was capable of detecting at least 0.4 pg/ml of IL-7 or IL-15.

### Elispot analysis

Human IFN-γ ELISPOT assays were performed as previously described[17] using capture and detection antibodies D1K and 7-B6-1 (10 µg/ml; Mabtech and Nacka), respectively. Autologous monocytes were pulsed at 10 ug/ml with the relevant peptide in parallel with an irrelevant peptide for each sample. Cryopreserved PBMCs from serial timepoint samples were plated at 1×10^5^ cells per well and co-cultivated with stimulator monocytes for 24 h. The plates were analyzed using an automated ImmunoSpot Analyzer (Cellular Technology). Results are presented as the mean number of spot forming cells/10^5^ PBMCs.

### Statistical analysis

Significance of variation between two groups was evaluated using a two-tailed paired t test, with p<0.05 considered significant(for Cytokine comparisons, CD4 levels, and Foxp3 analysis). The Wilcoxon signed rank test was used to compare T cell persistence before and after fludarabine. Linear regression for best-fit analysis, paired t tests, and Wilcoxon signed rank test were performed on a GraphPad Prism™ 4.0a software.

## Results

### Patient Demographics

Nine of the patients presented with cutaneous melanoma and one with anal mucosal melanoma (**Table 1**). Eight of ten patients had multiple sites of metastatic disease. Two patients had previously received stereotactic brain irradiation and at the time of study entry had no evidence of recurring CNS disease. All patients had failed previous therapy with conventional treatments for metastatic melanoma (IFN, high dose IL-2, biochemotherapy, or chemotherapy). Three patients had previously been treated on and progressed after earlier adoptive T cell therapy protocols targeting MART-1 and gp100 antigens[1].

**Table 1:** Patient Characteristics.

Pt	Age	Melanoma	Previous Therapy*	Disease sites†	LDH	Stage
1	48	cutaneous	Melacine, Chemotherapy	Bone, AD, LN, DU	↑	M1c
2	53	cutaneous	Biochemotherapy, ACT, XRT	Bone, SQ, LN	↑	M1c
3	49	cutaneous	Biochemotherapy, IL-2, IFN	LU, LN, PV, SQ	normal	M1c
4	71	cutaneous	Melacine, IFN, ACT	LU, spleen	normal	M1c
5	56	cutaneous	IL-2	LU	normal	M1b
6	59	anal	Biochemotherapy	LU	normal	M1b
7	56	cutaneous	IL-2, brain XRT	Brain, AD, DU	normal	M1c
8	36	cutaneous	IL-2, IFN, ACT	LU, LN	↑	M1c
9	67	cutaneous	Chemotherapy, IFN, brain XRT	Brain, LU, Liver	normal	M1c
10	28	cutaneous	Chemotherapy, XRT	LU, LN	normal	M1b

Staging per AJCC staging criteria (M1b metastasis to lungs, M1c metastasis to other visceral sites or elevated LDH).

* ACT, adoptive cellular therapy; XRT, radiation.

† AD, adrenal; DU, duodenal; LN, lymph node; LU, lung; PV, pelvic.

### Toxicities associated with Adoptive T cell therapy using melanoma-associated antigen specific CD8+ T cells and fludarabine conditioning

Patients were monitored for signs or symptoms of toxicity including autoimmune disorders since Mart-1, gp100, or tyrosinase are found in normal melanocytes and uveal tissue. No grade III or IV toxicity was observed[14]. All patients experienced fevers, mild nausea, and fatigue consistent with cytokine release syndrome beginning soon after T cell infusion and remitting spontaneously within 48 hours. One patient, #8, developed an erythematous rash around pigmented areas of skin on day 4 after his first infusion of MART-1 specific T cells. A skin biopsy revealed an intense lymphocytic infiltrate and immune mediated destruction of nevomelanocytes (data not shown) consistent with our previous description of T cell mediated vitiligo[18]. Patient #7 received steroids before his second infusion for progression of CNS disease; his second T cell infusion was not given. Patient #3 developed a grade II pneumonia six weeks after his fludarabine during a period of prolonged lymphopenia and was treated successfully with oral antibiotics.

### Clinical Responses

All patients at the time of T cell infusion had progressive metastatic disease. One patient received only one infusion of T cells. Of the nine patients who were able to receive both planned infusions, disease stabilization was observed in two patients for a period of 5.8 and 7.0 months, and one minor pulmonary response with disease progression at 11.0 months (**Table 2**). All other patients experienced progressive disease. Median progression free survival and overall survival was 2.1 and 9.7 months, respectively. Curiously, two patients (patients #5 and #8), whose disease progressed at initial re-staging developed delayed disease stabilization and had prolonged survival for 10.1 and 13.7 months after the initial T cell therapy. Such delayed responses following a period of apparent progression, have been observed following other immunotherapeutic strategies, such as anti-CTLA-4 therapy and may represent an early inflammatory response, followed by delayed disease remission[19].

**Table 2:** T cell persistence (Tp) *in vivo* as determined by tetramer analysis.

Pt	Antigen target	IL-2	INF#1 Tp (days)	INF#1 peak %tet/CD8	INF#2 Tp (days)	INF#2 peak %tet/CD8	Toxicity*	Response†	PFS (days)	OS (days)
1	Tyrosinase	No	5	0.29	13	0.40	F	PD	51	124
2	Gp100	No	4	0.11	6	0.18	F	PD	60	71
3	Mart-1	No	3	0.31	13	1.39	F, PNA	MR	330	535
4	Mart-1	No	5	0.84	27	2.32	F	PD	56	97
5	Tyrosinase	Yes	0	0.13	12	3.81	F	PD	73	412
6	Mart-1	Yes	6	0.20	6	5.25	F	SD	175	278
7	Gp100	Yes	38+	5.89	n/a	n/a	F, R	PD	n/a	96
8	Mart-1	Yes	24	7.98	63+	13.9	F, R	PD	63	351
9	Mart-1	Yes	0	0.088	2	0.26	F	PD	51	304
10	Mart-1	Yes	0	0.087	20	3.19	F	SD	209	313
	**Median**		**4.5**	**0.25%**	13.0**	**2.32%**			**63**	**291**

Comparison of T cell persistence between first infusion (INF#1) and second infusion (INF#2) shows a 2.9 fold increase (**p = 0.0078, Wilcoxon-signed rank). Patients #5 and #8 exhibited delayed disease stabilization.

* F, fever; PNA, pneumonia; R, rash.

† PD, progressive disease; SD, stable disease; MR, minor response.

OS, overall survival; PFS, progression free survival.

### Fludarabine leads to a decrease in CD4 lymphocyte count and increased plasma levels of homeostatically-regulated cytokines

**Figure 2:**
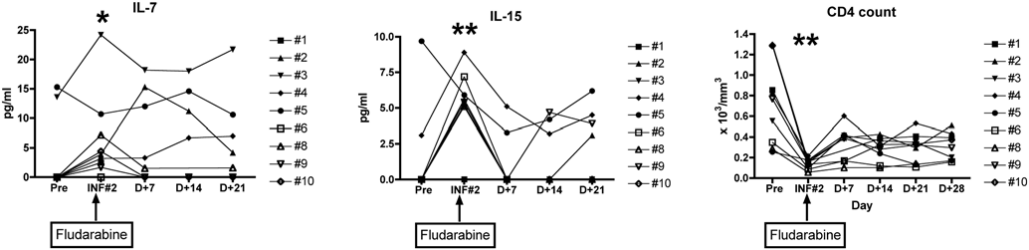
Plasma levels of IL-7 and IL-15 were measured at serial timepoints pre- and post fludarabine conditioning. CTL infusion #2 (INF#2) is administered two days after the final dose of fludarabine. *p = 0.053 comparing pre-fludarabine (pre) versus day of INF#2 (for IL-7) and **p<0.01 for pre- versus day of INF#2 (for both IL-15 and absolute CD4 levels).

Median pre-infusion (baseline) absolute CD4+ cell count was 770/mm^3^ (range 280–1350/mm^3^). CD4 counts declined to a median of 150 cells/mm^3^ (50–220) after fludarabine and remained below normal for at least four weeks (**Fig. 2**). Median CD4 count at D+28 was 250/mm^3^ (160–511), and in two cases remained <500/mm^3^ for up to 8 weeks after conditioning. As anticipated, this period of relative CD4 lymphopenia was accompanied by an elevation in IL-7 and IL-15 plasma levels (compared to baseline pre-infusion levels). Analyses for homeostatic cytokines IL-7 and IL-15 were obtained in serial plasma samples prior to the first T cell infusion, just prior to the second T cell infusion, and weekly thereafter. Median plasma IL-7 levels rose from undetectable levels at baseline to 2.9 pg/ml (0–24.2) at day 0 of the second infusion (p = 0.053). Median plasma IL-15 levels rose from undetectable levels at baseline to 5.5 pg/ml (0–8.9) at day 0 of the second infusion (p<0.01).

### Fludarabine lymphodepletion leads to a 3-fold increase in the persistence of adoptively transferred T cells

**Figure 3:**
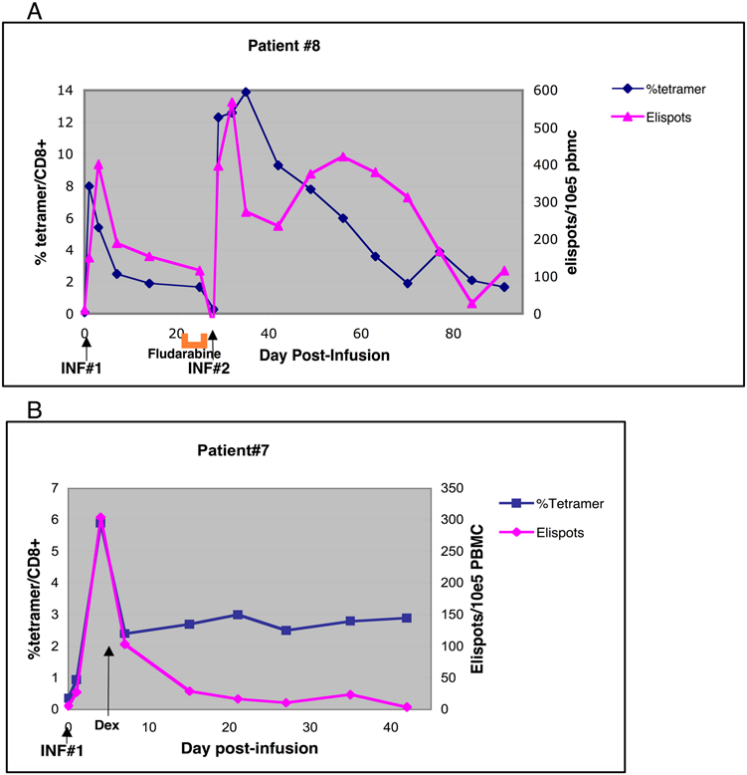
A. *In vivo* persistence of CTL clones as measured by tetramer+ CD8+ T cells from post-infusion, cryopreserved PBMCs. Superimposed are the correlative IFN-γ ELISPOT data. ELISPOT results are presented as the mean number of spot forming cells/10^5^ PBMCs. Shown is patient #8 who demonstrated prolonged persistence of Mart-1_27–35_ specific CTL. B. *In vivo* persistence and ELIspot data for patient #7 who received only one infusion of Gp100_154–162_ specific CTL clones. The long arrow indicates initiation of high dose dexamethasone (dex).

The *in vivo* survival of transferred T cells was determined by measuring the frequency of tetramer^+^ CD8 T cells in peripheral blood samples (see **Table 2**). The median *in vivo* persistence of transferred T cells was 4.5 days (range 0–38+) after the first T cell infusion and extended to 13.0 days (2–63+) after the second infusion (p = 0.0078, Wilcoxon signed rank). Overall, fludarabine improved the *in vivo* persistence of transferred T cells by an average of 2.9 fold. Tetramer^+^ CTL frequency peaked on days 1–3 post-infusion at a median of 0.41% (0.10–7.98) of CD8+ T cells after the first infusion and increased to 2.32% (0.18–14.1) after the second infusion. Three patients (Pt #5, 9, and 10) did not have an appreciable presence of transferred T cells after the first infusion in peripheral blood by our criteria, however, they clearly developed a measurable and significant tetramer^+^ population after fludarabine conditioning and second infusion. Among the three patients who had received therapy with CD8+ T cell clones on our previous T cell trial, there was no evidence of a residual infused T cell population at baseline. Patient #7 received only the first infusion, yet tetramer^+^ T cells were detectable at >2% of CD8+ T cells for at least 38 days. In patient #8, the presence of tetramer^+^ population was observable for at least 63 days at a level of >1% of CD8 cells (**Fig. 3**).

### Functional capacity of post-infusion CTL clones

We measured the functional capacity of adoptively transferred CTL clones to produce IFN-γ in response to *in vitro* antigen after T cell infusion. Sufficient PBMCs at serial timepoints post-infusion were available for functional analysis in six patients. Post-T cell infusion samples demonstrated a measurable population of IFN-γ producing cells responding to the cognate epitope by the ELISPOT assay (see example in **Fig. 3A**). The presence of these IFN-γ secreting cells paralleled the *in vivo* persistence of adoptive T cell clones except for one patient receiving steroid therapy (see below: patient#7). In general, approximately 30% of the infused CTL clones identified *in vivo* by tetramer analysis were capable of secreting IFN-γ by ELISPOT analysis (adjusted for CD8+/PBMC ratio). When these clones are tested *in vitro* under optimal conditions, a comparable fraction (25 to 30%) of T cells are producing IFN-γ at any one time suggesting that the infused clones retain functional responsiveness *in vivo*.

Patient #7 received high dose corticosteroids four days after his first T cell infusion for a recurrent CNS metastasis (**Fig. 3B**). He was immediately placed on high dose dexamethasone. While this did not apparently affect the numeric persistence of the transferred Mart-1 specific T cell clone, the *in vivo* effect of corticosteroids clearly diminished the capacity of the CTL clone to produce IFN-γ in response to antigen stimulation.

### Fludarabine lymphodepletion leads to a relative increase in Foxp3+ Regulatory T cells during recovery

**Figure 4:**
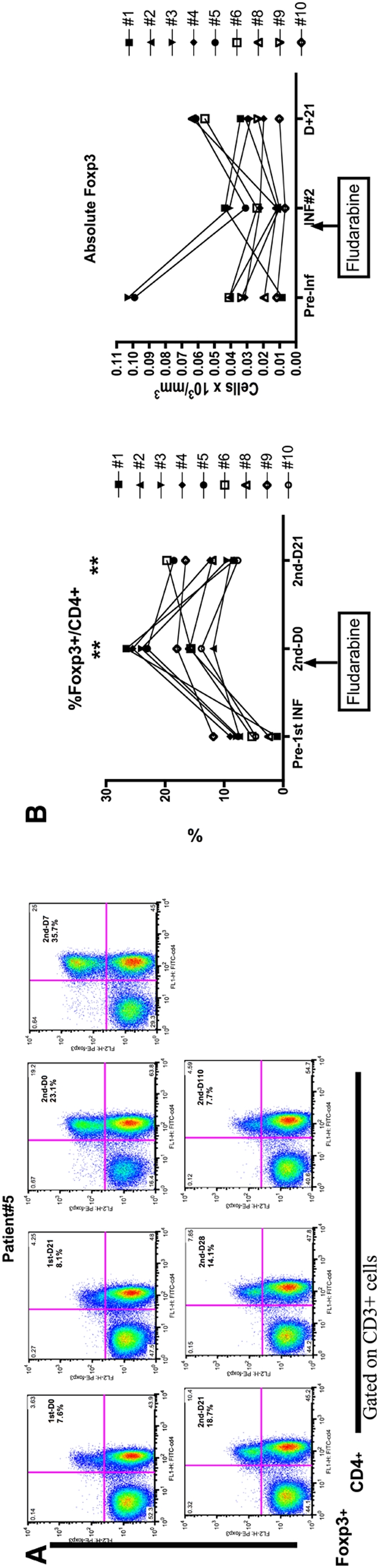
Fludarabine conditioning increases regulatory CD4+Foxp3+ T cells. A. Representative example (patient #5) of flow cytometric analysis for CD4+Foxp3+ T cells from pre and post-CTL infusion PBMC samples. Infusion number and post-infusion day is designated along with percentage of CD4+Foxp3+/CD4+ cells. B. The relative percentage and absolute CD4+Foxp3+ population is reported for the nine patients who received fludarabine. Samples are tested in duplicate with the average values reported. Comparisons between baseline Foxp3 levels vs. day 0 of INF#2 (**p<0.001) and D21 of INF#2 (**p<0.01).

**Figure 5:**
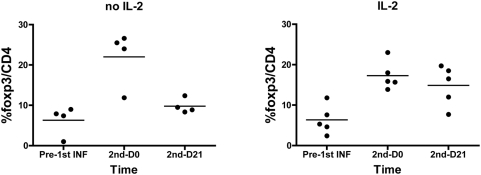
CD4+Foxp3+ levels are sustained in the presence of IL-2 (n = 5) compared to the absence of IL-2 (n = 4) at day 21 after CTL Infusion#2 (trend at p = 0.093, unpaired t-test).

Following fludarbine lymphodepletion, the percentage of CD4+ cells expressing Foxp3 rose from a median pre-therapy level of 7.4% (range 0.5–11.8%), to 18.0% (11.9–26.6%) just prior to the second T cell infusion (p<0.01), and remained elevated at 12.0% (7.72–19.7%) on day 21 after the second infusion (p<0.01) (**Fig. 4**). The first four patients on the protocol did not receive IL-2 supplementation. At Day 0 of the second T cell infusion, an increase in fraction of Foxp3+ cells is observed for all patients. However, by D+21, patients who did not receive exogenous IL-2 normalized the fraction of Foxp3+ cells to near baseline levels while those who received IL-2 experienced persistently elevated levels of Foxp3+/CD4+ ratio (trend at p = 0.093) (**Fig. 5**). Interestingly, while the relative percentage of Foxp3+ cells rose significantly following lymphodepletion, the absolute numbers of Foxp3+CD4+ cells remained relatively unchanged. There was no clear relationship between numeric or functional persistence of transferred T cells and the degree of Foxp3+/CD4+ population (p = 0.59).

Limited results of functional analysis from post-infusion PBMC samples of flow cytometry sorted CD4+CD127lo cells were explored in two patients (see **Fig. S1**). CD127lo cells contain the Foxp3 hi expressing subset and are readily flow-sorted [20]. Comparison of CD127lo and CD127hi cells from post-infusion PBMC samples in a mixed lymphocyte reaction with autologous responder and irradiated allogeneic stimulator cells demonstrate that CD127lo cells suppress thymidine incorporation in a proliferation assay by 36% in one patient and 18% in another (sorted to responder ratio of 0.3∶1, tested in duplicate) [20].

## Discussion

Use of *ex vivo* generated antigen specific T cell clones permits selection of a uniform population of well-defined cytotoxic T cell clones for infusion with known *in vitro* functional performance characteristics. In our previous studies, we have shown that adoptively transferred antigen-specific CD8+ CTL clones are detectable post-infusion, persist in the presence of IL-2 supplementation, are able to traffic to tumor sites, and effect anti-tumor responses[1]. In second generation studies, we capitalized on the use of CTL clones in a two-infusion intra-patient schedule that allows us to rigorously evaluate the influence of a given intervention (in this case, a pre-infusion conditioning regimen) on adoptively transferred T cells. In the present example, fludarabine was used for its predictable kinetics, low toxicity and capacity to elicit CD4 lymphopenia at a level we hypothesized to be sufficient to induce upregulation of homeostatic cytokines and reduction of inhibitory regulatory T cells, beneficial to transferred T cell survival.

Our studies reveal that fludarabine mediated lymphodepletion was modest (median CD4+ nadir ∼150cells/ul), but sufficient for the upregulation of detectable levels of homeostatic cytokines (IL-7 and IL-15) and enhancement in the duration of *in vivo* persistence of transferred T cells by almost 3-fold. IL-15 is well described as a homeostatic cytokine promoting CD8+ T cell survival and proliferation[4], [21]. It is not clear whether exogenous IL-2 is critical in lymphodepleted setting, however, since the four patients who did not receive exogenous IL-2 had similar duration of T cell persistence (median Tp post-fludarabine 13 vs 12 days).

Transferred T cells after fludarabine conditioning were detectable at high frequencies. In six patients, they comprised >2% of the CD8+ compartment in the peripheral blood from days 1–3 post-infusion. In two of the patients, (Patients 7 and 8), they were present in >1% frequency of CD8+ cells at day 38 and 63 after infusion. In our previous study (1), the median persistence of T cells in the presence of IL-2 was nearly 17 days compared to 4.5 days (without conditioning) and 13 days (post-fludarabine) in this present study. In contrast to our previous study, in order to administer identical T cells in the two infusions, T cells were cryopreserved until needed and thawed prior to infusion. Although viability of T cells immediately prior to infusion was excellent (>90%), this process may have affected the ultimate survival and proliferative ability of T cell clones.

This study was designed to reduce the interpatient variability by examining T cell persistence before and after administration of fludarabine. The median T cell persistence after the first infusion was 4.5 days. Only one patient had any detectable level of transferred T cells at the time of fludarabine adminstration (given on days 21–25 after first T cell infusion). Fludarabine rapidly resulted in lymphodepletion and elimination of any residual T cells in that patient (#8). At the time of the second T cell infusion, none of the T cells from the first infusion were detectable with tetramer monitoring. However, one limitation of this study is that we cannot fully rule out the possibility of a sub-detectable population from the first T cell infusion involved in altering the immunologic milieu for the second infusion. Because fludarabine rapidly and effectively depletes 80–90% of endogenous T cells, a cross-over effect from the first infusion is very unlikely.

Transferred T cell clones also remain functional after infusion, capable of producing IFN-γ in response to antigen specific stimulation with one exception. One serendipitous result of using a well-defined clonal T cell population for adoptive therapy, was a dramatic *in vivo* demonstration of corticosteroid's deleterious effects upon the functional capacity but not survival of CD8+ T cells in a patient who received high-dose dexamethasone shortly after his T cell infusion. Activation of the glucocorticoid receptor on T cells is known to selectively inhibit expression of IFN-γ and other Th1 cytokines, possibly by direct interaction with the Th1-specific transcription factor T-bet[22]. While this has been shown *in vitro*, this example would be the first demonstration that corticosteroids can modulate the functional capacity of adoptively transferred human effector T cell clones *in vivo*, underlining the importance of evaluating both numeric and functional persistence when tracking T cells *in vivo* for efficacy *and* toxicity.

In addition to upregulating homeostatic cytokines, fludarabine conditioning was postulated to reduce regulatory Foxp3+ CD4+ cells (Treg). Treg cells, characterized as CD4+/CD25hi/foxp3+ are known to be expanded in a variety of hematologic[23]as well as solid tumor malignancies[6], [24], [25]. Treatment of CLL patients with fludarabine was associated with a decreased frequency of regulatory CD4+CD25^hi^ cells[26]. However, in that disease, fludarabine has a direct anti-tumor effect in CLL, and the decline of Treg population may simply reflect a smaller tumor burden with therapy. In contrast, fludarabine has no direct anti-melanoma effect. We observed, surprisingly, an increase in the proportion of Foxp3+ cells within the recovering CD4+compartment after fludarabine lymphodepletion. This is the first time fludarabine has been shown to enhance Foxp3+ expressing cells. Treg cells may be more resistant to apoptosis[27] and/or recover from lymphodepletion faster than their non-regulatory counterparts. Peripheral expansion of CD4+CD25^hi^ Treg *in vivo* is regulated by both IL-2 and lymphopenia[28]. Thus, there is concern regarding the growth enhancing effect of IL-2 administration during lymphopenia. Our data showing protracted elevation of the Treg population in the presence of IL-2 and lymphodepletion would support this contention.

Furthermore, there are safety concerns regarding fludarabine when combined with other severe lymphodepleting modalities. Fludarabine may contribute to a number of serious complications observed in other studies[29] including opportunistic infections, cortical blindness, and prolonged immunosuppression, the latter ultimately being counterproductive by inhibiting endogenous CD4+ “help.”

While the clinical responses in this trial are modest, this treatment was undertaken in a very heavily pretreated population. Three of the patients in this protocol had previously received adoptive T cell targeted therapy on other trials and had progressed. Median overall survival for all ten patients is 9.7 months. This fares very favorably compared to historical control of ∼4 months for patients with refractory metastatic melanoma[30]. Three of the nine patients who received both T cell infusions achieved minor or stable disease lasting 5.8–11.0 months. Curiously, two patients exhibited delayed disease stabilization after initial progression and had prolonged survival for 10.1 and 13.7 months after T cell therapy. This could reflect delayed immunologic responses and has been observed following other immunotherapeutic strategies, such as anti-CTLA-4 therapy[19]. It may represent early inflammatory response described as radiographic progression followed by delayed disease responses.

This first study of involving lymphodepletion and CD8+ T cell clones was designed to limit toxicity of lymphodepletion while balanced to promote homeostatic cytokines and reduce regulatory T cells. We acknowledge that higher degrees of lymphodepletion may increase the availability of homeostatic cytokines and further reduce regulatory T cells. However, the prolonged CD4 lymphopenia observed with fludarabine may be counterproductive from the depletion of helper CD4+ cells. Other lymphodepleting agents, such as cyclophosphamide (when given in high doses), result in greater, but transient degree of lymphodepletion and may provide a selective advantage.

Potential opportunities to further improve T cell persistence and clinical response, include, achieving a greater degree of lymphodepletion and enhanced homeostatic cytokine availability with the use of high dose cyclophosphamide, selective depletion of regulatory CD4 T cells[31], augmentation with high dose IL-2, and increased availability of CD4 help. The latter may be achieved through the transfer of antigen specific CD4+ T cells in tandem with CD8+ T cell clones to enhance the effector function/proliferative ability of CD8+ cell, and independently provide anti-tumor activity. Clinical trials including combination of these elements are currently underway. In summary, adoptive T cell therapy with CTL clones is safe and provides a potentially robust instrument for dissecting the requirements for a successful T cell strategy. Use of antigen specific T cell clones remains a viable strategy for treatment of metastatic melanoma, but further step-wise optimizations to improve T cell persistence and address regulatory T cells will be important components in enhancing anti-tumor efficacy.

## Supporting Information

Checklist S1 (0.05 MB DOC)

Flow Diagram S1 (0.03 MB DOC)

Protocol S1 (0.76 MB PDF)

IRB approval S1 (0.23 MB PDF)

Figure S1 Fludarabine induced regulatory T cells have suppressive capability. Functional analysis from post-infusion PBMC samples of flow cytometry sorted CD4+CD127lo cells. CD4+CD127lo and CD4+CD127hi cells were sorted by flow cytometry in two patients 14 days after T cell infusion. We compared CD127lo and CD127hi subsets in a mixed lymphocyte reaction with autologous responder and irradiated allogeneic stimulator cells. CD127low cells suppress thymidine incorporation in a proliferation assay by 36% in one patient and 18% in another (sorted to responder ratio of 0.3∶1, tested in duplicate).
(0.23 MB TIF)
