# Supplementary material for: Fludarabine Modulates Immune Response and Extends In Vivo Survival of Adoptively Transferred CD8 T Cells in Patients with Metastatic Melanoma
Source: PLoS One. 2009 Mar 9;4(3):e4749. doi: 10.1371/journal.pone.0004749 (PMC2650617; doi:10.1371/journal.pone.0004749)
Supplement: Flow Diagram S1 — (0.03 MB DOC) [file pone.0004749.s002.doc]

**The Consort E-Flowchart Aug. 2005**

Assessed for eligibility(n=10 )

Excluded (n=0 )

Not meeting inclusion criteria

(n=0 )

Refused to participate

(n= 0 )

Other reasons

(n= 0 )

**Allocation**

**Analysis**

**Follow-Up**

**Enrollment**

Analyzed (n=10 )

Excluded from analysis (n= 0 )

Give reasons

Lost to follow-up (n= 0 )

Give reasons

Discontinued intervention

(n= 0 )

# Give reasons

Allocated to intervention

(n= 10 )

Received allocated intervention

(n= 10 )

Did not receive allocated intervention

(n= 0 )

Give reasons

Lost to follow-up (n= n/a )

Give reasons

Discontinued intervention

(n= n/a )

Give reasons

Allocated to intervention

(n= n/a )

Received allocated intervention

(n= n/a )

Did not receive allocated intervention

(n= n/a )

Give reasons

Analyzed (n= )

Excluded from analysis (n= )

Give reasons

Is it Randomized? No
