# Supplementary material for: Fludarabine Modulates Immune Response and Extends In Vivo Survival of Adoptively Transferred CD8 T Cells in Patients with Metastatic Melanoma
Source: PLoS One. 2009 Mar 9;4(3):e4749. doi: 10.1371/journal.pone.0004749 (PMC2650617; doi:10.1371/journal.pone.0004749)
Supplement: Protocol S1 — (0.76 MB PDF) [file pone.0004749.s003.pdf]

FRED HUTCHINSON CANCER RESEARCH CENTER
UNIVERSITY OF WASHINGTON SCHOOL OF MEDICINE
CHILDREN'S HOSPITAL AND REGIONAL MEDICAL CENTER
Previous Version: 12/23/05
Current Version: 04/19/06

1. TITLE

Phase I Study To Evaluate The Safety Of Cellular Adoptive Immunotherapy Using Autologous CD8$^+$ Antigen-Specific T Cell Clones Following Fludarabine Lymphodepletion For Patients With Metastatic Melanoma

Investigators:

Cassian Yee, MD	Associate Member, FHCRC,	667-6287
John Thompson, MD	Associate Member, FHCRC, Associate Professor, UW	288-2044
David Byrd MD	Associate Professor, UW	543-7512
Raymond Yeung, MD	Associate Professor, UW	616-6408
Herschel Wallen, MD	Senior Fellow, FHCRC	667-5897

Trial Coordinator: Lisa Schirmer 667-1539

Statistician: Ted Gooley PhD, Associate Member, FHCRC.

Emergency 24-hour phone: 206-598-8902 FAX: 206-598-4034

2. INTRODUCTION

This protocol proposes to examine the safety and duration of *in vivo* persistence of adoptively transferred autologous antigen-specific CD8$^+$ CTL clones administered following fludarabine lymphodepletion in patients with melanoma. The cells will be infused into patients with metastatic (Stage IV) melanoma. These patients historically have a very poor prognosis with a median survival of less than 7 months (1). A secondary objective of this study is to assess the *in vivo* antitumor effect of the infused autologous antigen-specific CD8$^+$ CTL.

2.1 Immunotherapy of Malignant Melanoma

2.1.1 Introduction

The incidence of malignant melanoma has increased during the past decade at a rate faster than any other cancer and will have reached > 1 in 90 by the year 2000 (2). Malignant melanoma currently afflicts 44,000 people each year. The median age of patients with melanoma is 45 years and thus, the loss of productive years is one of the most significant among cancers (2). Chemotherapy and radiation therapy represent the standard treatment for patients with extensive disease at presentation or with recurrent disease after surgical resection, but response rates are low and the responses are not usually durable. As a result many patients are treated palliatively or are enrolled in investigational studies (1).

2.1.2 Therapy of Melanoma with Lymphokine-Activated Killer Cells

Melanoma is one of a handful of malignancies that can spontaneously regress without treatment. These rare events have suggested a potential role of the host immune system in promoting tumor regression. The availability of recombinant interleukin 2 (IL-2) and the demonstration that lymphocytes cultured in high concentrations of lymphokines acquired the ability to lyse in a non-MHC-restricted fashion a variety of tumor targets including melanoma cells (3, 4) led to trials attempting to augment the patient's own immune system by the administration of IL-2 and/or autologous lymphokine-activated killer (LAK) cells that have been propagated *ex vivo* from the peripheral blood. Up to 10^{11} LAK cells have been administered in a single intravenous infusion to cancer patients without toxicity that was attributable to the infused cells, demonstrating the safety of therapy with large numbers of *in vitro* activated lymphocytes (3, 5, 6). The toxicity that was observed in these trials was attributed solely to the systemic effects of IL-2.

2.1.3 Tumor Infiltrating Lymphocyte (TIL) Therapy of Melanoma

Recent studies have examined the administration of lymphocyte populations isolated in vitro from tumor specimens on the assumption that such cells found near the tumor site are more likely to be tumor-reactive. These TIL were isolated by mincing tumor samples and culturing the eluted lymphocytes with very high doses of IL-2. In a murine sarcoma model such TIL cells have been shown to be 50-100 times more effective than LAK cells in eradicating tumor micrometastases (7). TIL lines generated in this manner appear to function either as T cells with lytic specificity for autologous but not allogeneic tumor targets, or as LAK cells and lyse both autologous and allogeneic tumor targets(8-10) . Adoptive transfer of up to 1011 TIL alone has not been associated with significant toxicity, and administration of TIL cells with concurrent systemic IL-2 has resulted in toxicities that are attributable to the IL-2(11, 12) . However, clinical trials involving the use of LAK cells or TIL cells in the treatment of patients with metastatic melanoma have met with only modest success in spite of the very high numbers of cells infused. The observed limited efficacy likely reflects several variables, including poor definition and quantitation of the effector cell that is presumably present in the heterogeneous TIL population, poor localization of these effector cells to the site of the tumor, poor in vivo function and survival of transferred TIL following in vitro growth in extremely high doses of IL-2 and failure of all tumor cells to express the undefined target antigens recognized by the TIL effector cells.

2.2 Immunotherapy of Melanoma using Antigen-specific T cells

2.2.1 Potential Tumor Rejection Antigens Recognized by CD8+ CTL

Although murine studies have demonstrated the ability of CD8+ CTL clones, raised against specific tumor antigens, to eradicate established tumors in vivo (13) the use of antigen-specific CD8+ CTL for the treatment of malignancies in human trials has not been implemented largely due to the difficulty in identifying useful tumor target antigens. Recently, however, work pioneered by Thierry Boon's laboratory in Brussels, and subsequently adapted by others, has resulted in the identification of potential tumor rejection antigens using tumor cell lines derived from melanoma patients (14,

15). Tumor-reactive CTL clones were generated using autologous tumor cells as stimulator cells, and these CTL were then used to screen antigen-negative targets transfected with pools of DNA derived from a genomic or cDNA library isolated from the tumor cell line. The tumor DNA in transfectants lysed by these CTL was recovered, resulting in the identification of several different melanoma antigen(14, 16, 17). These antigens can be divided into two groups: 1) cancer-testis or CT antigens that are expressed only in tumor and germinal tissue such as testis and have been designated: MAGE-1, MAGE-3, GAGE and NY-ESO-1; and 2) 'differentiation antigens' that include proteins associated with melanin synthesis: tyrosinase, MelanA, gp100 and gp75(18). The function of the cancer-testis (CT) proteins is not known but may have a role in cell cycle(19). The ‘differentiation antigens’ function in melanin synthesis and are also expressed in normal pigment producing cells such as melanocytes (17, 20-22).

2.2.2 Isolation and Expansion of Antigen-specific T Cell Clones from the Peripheral Blood of Patients with Melanoma.

In previous studies, we have been able to isolate and expand from the peripheral blood of normal donors and melanoma patients, antigen-specific CTL clones that recognize tumor cells. Several peptides have been identified which represent the immunogenic epitopes of MART-1/Melan-A, gp100 and tyrosinase. We have used these peptides to successfully generate antigen -specific CTL clones that recognize antigen-positive tumors in vitro. The details of T cell generation and expansion are previously published (23-27) and are detailed in Appendix A. Briefly, this was accomplished in the following manner - autologous dendritic cells were pulsed peptides corresponding to immunogenic epitopes, prior to in vitro stimulation of CD4-depleted peripheral blood mononuclear cells (PBMCs) in 48-well plates. This was repeated two more times at one week intervals using thawed adherent mononuclear cells pulsed with peptide. Low dose (10U/ml) IL-2 was added after the 2nd and 3rd stimulation to promote expansion of reactive T cells expansion.

CD8+ T cells from cultures stimulated using autologous antigen presenting cells pulsed with peptides were then cloned by limiting dilution using anti-CD3 monoclonal antibody (30ng/ml) and 50 U/ml of IL-2. Ten to 14 days later, clones were selected and tested for antigen-reactivity with a micro-chromium release assay. Antigen-specific CTL clones were expanded 4 to 5 $\log_{10}$ in number by repetitive cycles of stimulation with anti-CD3 mAb in cultures supplemented with irradiated feeder cells. T cells isolated in this manner express the high affinity IL-2 receptor following TCR engagement, proliferate in response to low doses of IL-2 in vitro (< 10 U/ml), acquire a morphologic and phenotypic activation phenotype, and revert to typical small, resting lymphocytes in vitro 12-16 days after stimulation. They therefore appear to retain the physiologic properties of memory effector T cells present in vivo.

2.2.3 Potential toxicity associated with the infusion of antigen-specific CTL.

One potential problem with infusing large numbers of antigen-specific CD8+ CTLs is that such T cells may recognize antigen expressed by normal tissues. Antigen is expressed in some cells derived from the neural crest such as melanocytes of

the skin and choroid and retinal cells. The pigmented tissues of the eye may be protected from potential toxicity since the subretinal space and vitreous cavity are considered immunologically privileged sites by virtue of physical barriers and immunosuppressive factors such as TGF-beta, alpha-MSH and other low molecular weight proteins present in these sites (28-30).

In humans, there have been reports of patients treated with immunomodulators such as gamma-interferon or IL-2 in whom the appearance of vitiligo parallels their response to therapy (31). Patients with metastatic melanoma have also been treated with infusions of large numbers of (up to 1011) CD8+ TIL cells that were subsequently shown to be reactive to antigens (gp100, Melan-A and antigen) shared by melanomas and normal melanocytes. Although vitiligo was observed in a small proportion of these patients, there was no evidence of visual or neurologic toxicity (32). More recently, we reported on the development of a targetoid rash surrounding normal pigmented skin cells on a patient who received MART-1 specific CD8+ T cells. The infused T cell clone was retrieved from these sites of inflammation. (26). The rash resolved without sequelae, and the patient did not develop evidence ocular or neurologic toxicity .

2.3 Adoptive T Cell Therapy for CMV disease using CMV-specific CD8+ CTL clones.

The safety of adoptively transferring antigen-specific CTL clones in humans and the in vivo persistence of these clones have been examined in bone marrow transplant patients who received CMV-specific CTL clones. Studies in our Program in Immunology at the Fred Hutchinson have demonstrated that the reconstitution of endogenous CMV-specific T cell responses following allogeneic BMT correlates with protection from severe CMV disease(33, 34). CD8+ CMV-specific CTL clones were generated from CMV seropositive HLA-matched sibling donors, expanded and infused into sibling BMT recipients at risk of developing CMV disease. Fourteen patients have been treated with four weekly escalating doses of these CMV-specific CTL clones to a maximum T cell dose of $10^9/m^2$ without any attendant toxicity. Peripheral blood was harvested from patients immediately prior to, and 2 days following each infusion and 1, 2, 4, 8 and 12 weeks after completing the infusions. The recoverable CMV-specific CTL activity increased after each successive infusion of CTL clones, and persisted at least 12 weeks after the last infusion as assessed by PCR and sequencing of the TCR genes expressed in the transferred and recovered CMV-specific CTL . No patients developed CMV viremia or disease(35). These results demonstrate that CMV-specific CD8+ CTL clones can be adoptively transferred safely to BMT recipients and can persist in vivo as functional effector cells that may provide protection from CMV disease.

2.4 Adoptive T Cell Therapy for Metastatic Melanoma Using Antigen-specific T Cells

2.4.1 Adoptive T Cell Therapy – CD8+ T Cell Clones

We recently completed a Phase I study to evaluate the safety, in vivo persistence and efficacy of adoptively transferred CD8+ T cell clones targeting the

tumor-associated antigens, MART1/MelanA and gp100 for the treatment of patients with metastatic melanoma (27). Four infusions of autologous T cell clones were administered, the first without IL-2 and subsequent infusions with low-dose IL-2 for 14 days. Forty-three infusions of MART1/MelanA-specific or gp100-specific CD8+ T cell clones were administered to 10 patients. No serious toxicity was observed. We demonstrated that the adoptively transferred T cell clones persisted in vivo in response to low-dose IL-2, preferentially localized to tumor sites and mediated an antigen-specific immune response characterized by the elimination of antigen-positive tumor cells, regression of individual metastases and minor, mixed or stable responses in 8 of 10 patients with refractory, metastatic disease for up to 21 months (27).

In the absence of co-administered IL-2, the median in vivo survival of T cells in this study was about 7 days. When T cell infusions were given with low dose IL-2 (250,000 U/m2 s.c. twice daily) for 14 days, median T cell survival was extended to > 16 days. Attempts to enhance T cell survival beyond this period by extending IL-2 administration beyond 2 weeks will likely not be feasible due to the induction of counterregulatory mechanisms following prolonged IL-2 administration (36-38). Indeed, in this study, T cell survival began to decline by day 10, before the 14-day course of IL-2 was completed

Rosenberg and colleagues recently reported on the use of adoptively transferred autologous CD8+ T cell clones in patients with melanoma and showed that transferred T cells failed to persist (even after nonmyeloablative conditioning) and produced no clinical effect (39, 40) in contrast to our own results demonstrating sustained in vivo persistence and evidence of anti-tumor immunity. This discrepancy underlines the hypothesis that the manner in which effector cells are generated in vitro may be critical for their in vivo function. In the studies performed by Rosenberg's group at the NIH, T cells specific for the gp100-derived G209 epitope were isolated from patients previously immunized with a modified G209 peptide (G209M) and had failed therapy. Unfractionated peripheral blood mononuclear cells from these patients were stimulated in vitro by the addition of G209M peptide followed by high doses of IL-2 (1000 U/ml). Clones that were isolated were expanded and infused into autologous patients (who had previously failed G209M vaccination) together with high doses of IL-2 (72,000 to 720,000 U/kg three times daily) (39, 40).

By comparison, in our studies, gp100 or MART-1 specific T cells were isolated from non-vaccinated patients by cyclical antigen stimulation using autologous peptide-pulsed dendritic cells in the presence of low-doses of IL-2 (25-50 U/ml). As a consequence, the T cells that were generated expressed the high affinity IL-2 receptor upon antigen stimulation and continued to respond to low-dose IL-2 in vivo (250,000 U/m2 twice daily - a total IL-2 dose more than 15 times lower than the lowest dose used in the NIH studies).

2.4.2 Adoptive T Cell Therapy – TIL Following Lymphodepletion

Significant tumor regression and clinical responses were reported more recently by Rosenberg and colleagues at the NIH in some patients receiving in vitro expanded tumor-infiltrating T cells and high-dose IL-2 following nonmyeloablative conditioning with cyclophosphamide (60 mg/kg for 2 days) and fludarabine (25 mg/m2 for 5 days) (41). Among 13 patients receiving non-uniform populations of CD4 and /or CD8 T cells of various antigen-specificities (MART-1, gp100, tyrosinase or others), in

two cases, over 90% of the reconstituted T cell repertoire was found to be comprised of the infused melanoma-specific T cells. It was clear that in these patients, a transferred clonal T cell population infiltrated tumor sites and induced major regressions. However, this strategy was accompanied by serious toxicities related to high-dose IL-2 therapy (although not as severe as in immune replete patients) and profound immunosuppression, including the development of EBV-lymphoma in two patients ((41) & personal communication). Although dramatic responses were observed, the phenotype and specificity of effector cells required to induce such a response, in general, could not be defined given the varying compositions of the cell populations infused (41). Additionally the influence of high-dose IL-2 alone (in the absence of transferred effector cells) in the setting of immunodepletion is not known.

2.4.3 The Lymphopenic Environment And Anti-Tumor Immunity

Induction of lymphopenia in murine models by sublethal total body irradiation has been shown to lead to tumor regression in some instances (42-44). Lymphopenia results not only in homeostatic expansion of naïve and memory T cells (45), but may also be critical for T cell homing to tumor. When transferred into sublethally irradiated lymphopenic mice, autologous T cells infiltrate extensively into tumor and inhibit tumor growth (44, 46). In adoptive therapy studies, transferred memory T cells from tumor-sensitized mice are capable of eradicating disseminated leukemia and providing long-term protection but only when mice were pre-treated with cyclophosphamide (cyclophosphamide alone in these mice failed to protect). Whether exposure to lymphocyte depleting regimens (i.e. TBI, cyclophosphamide or fludarabine, etc) provides "space" (47), promotes homeostatic proliferative signals (48, 49) or reduces the population of host or tumor-derived regulatory cells (e.g. CD4+ CD25+ T cells) (50, 51) (52, 53), the evidence suggests that lymphodepletion can induce a significant anti-tumor response that is mediated by antigen-specific T cells.

2.4.4. Use of Fludarabine in CLL and in Metastatic Melanoma

Fludarabine (Fludara, Berlex Inc.) is a purine analog of adenine that inhibits DNA synthesis by impairment of ribonucleotide reductase and DNA polymerase alpha and induces lymphopenia in greater than 30% of patients treated with a standard 5 day course of fludarabine at 25 mg/ m^2 (54). It has significant effects in a wide range of indolent lymphoproliferative diseases including CLL, low grade non-Hodgkin's lymphoma, and Waldenström's macroglobulinemia (55-59), but has no effect on solid tumors, including malignant melanoma (60-62).

Lymphopenia is characterized by a CD4 > CD8 T cell depletion that results in lymphocyte counts falling to 30-40% of baseline or less that is sustained for periods of several weeks to months and is associated with T cell dysfunction-related infections (63, 64). Approximately 25% of patients receiving a 5-day course of treatment will develop an infection that is generally treatable. Patients receiving only a 3-day course of fludarabine exhibit a significantly lower incidence of infection per treatment course (14%) without a significant decrease in anti-lymphoproliferative effect (65).

2.4.5. Use of Interleukin-2 in Patients with Metastatic Melanoma

IL-2 promotes the proliferation of immune effector cells such as T cells and NK cells by binding to high and intermediate affinity surface receptors. Initial studies using high dose IL-2 for the treatment of metastatic melanoma and renal cell carcinoma have demonstrated responses in 3 - 24% of patients with rare durable remissions. These

doses, in the range of 18 to 28 x 10^6 U/ m^2 every 8 hours resulted in significant morbidity as a result of capillary leak syndromes and occasional mortality (41). In contrast, patients receiving low-dose recombinant IL-2 at doses up to 3 x 10^6 U/m^2 for periods of 1 month or more do not experience significant toxicity (WHO classification, Grade III or IV) (42-44). While such doses have seldom resulted in clinical response, doses as low as 100,000 U have been demonstrated to induce cellular immunologic effects in vivo while doses greater than 10^6 U/m^2 were associated with systemic symptoms of malaise and myalgias. Following subcutaneous administration, IL-2 exhibits a half-life of between 3 to 12 hours, sustained serum levels of 10- 25 U/ml, and receptor saturating serum concentrations of 22 pM after an injection of 250,000 U/m^2 (45-48). Administration of 3 x 10^6 U/m^2 of IL-2 for up to 21 days for the treatment of melanoma results in response rates of 0 to 9% (49). Therefore, at doses used in this trial, of 2 x 10^6 U/m^2/d or less, it is unlikely that patients will receive significant benefit from the IL-2 alone, rather, the IL-2 is administered in a manner designed to promote persistence of infused T cells in vivo.

2.4.6. Use of Interleukin-2 in Adoptive T Cell Therapy

Analysis of T cell persistence following 22 infusions of CD8+ T cell clones without IL-2 demonstrated a pattern of T cell survival characterized by an average increase in T cell frequency on Day 1, from < 1/20,000 to 1/480 PBMCs or > 1% of all CD8+ T cells, followed by a steady rapid decline to 1/1500 by Day 7 and < 1/20,000 by Day 14. The median survival of CD8+ T cells given without IL-2 was 6.56 +/- 0.82 days. To extend the period of in vivo persistence, the study was modified to include four unmodified T cell infusions three weeks apart, the 2nd, 3rd and 4th infusions being accompanied by a 14-day course of low-dose IL-2 following each infusion (at doses of 0.25, 0.50 and 1.0 MU/m^2 s.c. twice daily). These doses are 50- fold less than the high doses of IL-2 administered for the treatment of melanoma, do not mediate the toxicity associated with high-dose infusions, and by itself, has no anti-melanoma effect. Analysis of T cell persistence from 12 infusions of CD8+ T cell clones given with low-dose IL-2 demonstrated a pattern of T cell survival characterized by an increase in frequency to 1/490 PBMCs or > 1% CD8+ cells in the peripheral blood on Day 1 that was sustained at an average of 1/890 PBMCs until Day 14 at which time, IL-2 was discontinued and T cell frequency fell to < 1/5700 by Day 21. The average median survival of CD8+ T cells following an infusion of T cells with IL-2 was 16.78 +/- 1.6 days which was significantly longer than that observed in infusions administered to the same patient without IL-2.

2.5 Rationale and Proposed Study of Adoptive T Cell Therapy for the Treatment of Patients with Metastatic Melanoma

The proposed protocol evaluates an approach to facilitate in vivo persistence and expansion of transferred CD8 T cells based on the observation that T cells undergo homeostatic proliferation and expansion in a lymphodepleted host, possibly as a result of multiple factors, including an increase in circulating cytokines, such as IL-7 and IL-15. While the study reported by Rosenberg et al demonstrated that adoptive transfer of autologous T cells following immunodepletion with high-dose cyclophosphamide and fludarabine resulted in vivo T cell expansion and in some cases, virtual replacement of the entire peripheral T cell repertoire with the infused clone, it is not clear that such a

skewed reconstitution of the immune system is required for anti-tumor effect nor that such a profound level of immunodepletion which was accompanied by serious toxicities, is necessary (or essential) , given that a less severe relative lymphopenia can also provide homeostatic proliferative signals to T cells. Fludarabine at 25 mg/m^2 for 3-5 days is known to induce Grade 2-3 lymphopenia (a 30-50% decrease in lymphocyte counts) an effect that is sustained for approximately 4-6 weeks. While some risk associated with lymphopenia is occasionally observed in patients receiving multiple cycles of fludarabine therapy, the degree of immunocompromise is not nearly as severe as the cyclophosphamide/fludarabine conditioning.

This trial will also further delineate the requirements for successful immunotherapy through the adoptive transfer of a uniform population of antigen-specific CD8+ T cell clones so that the reasons for success or failure can be more clearly defined. T cells will first be given in the absence of a conditioning regimen, and then, following a 5 day course of fludarabine. Intrapatient analysis of T cell persistence in vivo between the two infusions can be directly compared and the contribution of fludarabine-induced lymphopenia to extending in vivo survival of adoptively transferred T cells evaluated.

2.6 Initial Results from Protocol 1796 "Fludarabine Lymphodepletion in Adoptive T Cell Therapy"

Four patients have been treated on Protocol 1796 on the original protocol which included the administration of fludarabine as a lymphodepleting agent before the second T cell infusion but no inclusion of IL-2 with either the 1st or 2nd infusion. As of June, 2004, analysis of the data after treating the first 4 patients on this study has demonstrated that:

1. there are no safety concerns (i.e., no serious adverse events)
2. in vivo persistence was prolonged slightly from median of 7 to 23 days

This increase is less than expected given the anticipation that prior lymphodepletion resulting in increased serum IL-7 and IL-15 levels would result in survival for several weeks and even in vivo expansion which was not observed in the clinical trial. In fact, the increase is only slightly increased compared to a 2 week course of low-dose IL-2 (median survival of 16.9 days). Published studies performed in murine models demonstrate that even with prior depletion (e.g. with cytoxan), CD8 T cell persistence may not be prolonged unless IL-2 is also given. In this case, the use of fludarabine also ablated any potential CD4 help from the peripheral blood and, alone, did not appear to be sufficient to provide prolonged in vivo persistence. Based on these observations and previously published studies using other lymphodepleting agents in combination with IL-2, low-dose IL-2 (at a dose previously shown to be safe and effective in prolonging T cell survival in vivo) will be incorporated into the design of the study. In this study, a 14-day course of low-dose IL-2 following both the first and second T cell infusions will be administered. Since fludarabine lymphodepletion precedes only the second T cell infusion, any differences in T cell survival can be attributed to lymphodepletion alone.

3. OBJECTIVES

3.1 PRIMARY OBJECTIVES

3.1.1 Assess the safety and toxicity of cellular adoptive immunotherapy in melanoma patients using autologous CD8+ antigen-specific T cell clones following fludarabine conditioning

3.1.2 Determine the duration of in vivo persistence of adoptively transferred CD8+ antigen-specific CTL clones without and with prior fludarabine-mediated lymphodepletion.

3.2 SECONDARY OBJECTIVE

3.2.1 Evaluate the antitumor effect of adoptively transferred CD8+ antigen-specific CTL clones following fludarabine-mediated lymphodepletion

4. STUDY DESIGN

This will be a single center, phase I, open-label, nonrandomized study in 12 individuals with metastatic melanoma (Stage IV disease). Antigen-specific T cells targeting one of the A2 or A3-restricted epitopes of the melanoma-associated antigens listed in Table 1 (Appendix A) will be generated in vitro.

Patients will receive T cell infusions according to the following schedule:

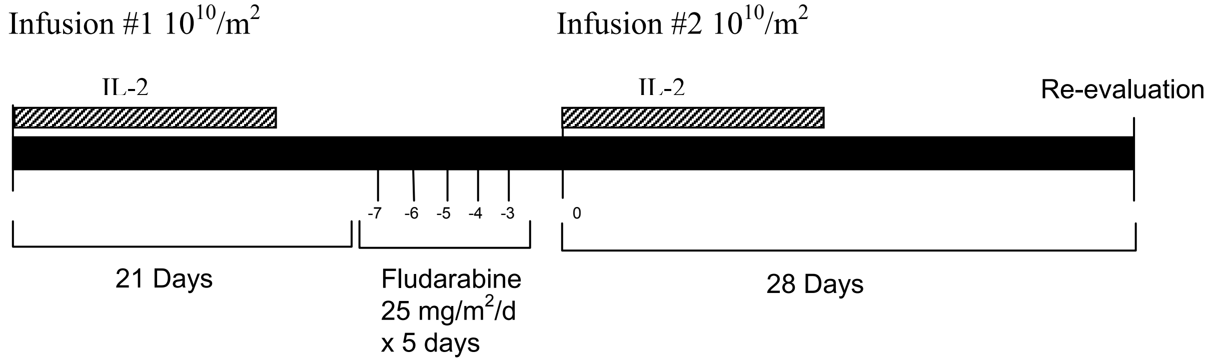

Antigen-specific CD8+ T cell clones will be administered at a dose of 10^{10} cells/m^2 for both Infusions #1 and #2. The median survival of transferred antigen-specific CTL clones in the presence of low-dose IL-2 (250,000 U/m^2) is approximately 17 days. By the end of 21 days, the frequency of transferred CTL is < 0.1% of the total CD8+ population. Fludarabine 25 mg / m^2 will be administered intravenously daily for 5 days prior to the second infusion of antigen-specific CD8+ CTL clones which will also be administered in the presence of low-dose IL-2. Four weeks after the second infusion, patients will be re-evaluated clinically and radiographic to determine the response to therapy. Serial blood draws during this time will allow us to determine if fludarabine lymphodepletion contributed to a prolongation of the median T cell survival beyond the expected 17 days seen with low-dose IL-2 alone.

If serious toxicity related to the T cell infusions is observed following infusion of CTL clones, , a regimen of corticosteroids will be given, and no further T cell infusions will be administered to the patient.

Subjects will be carefully monitored at regular intervals for a total of approximately 9 months following the last infusion. Evaluations after this time will be carried out by the referring physician who will be asked to notify us of the patient's progress. Peripheral blood from the patient will also be drawn. Patients receiving T cells will have up to approximately 4 tablespoons of blood drawn immediately prior to each infusion, 24 hours after each infusion, and then at specified intervals for 4 weeks from the last infusion (Appendix D). Samples will be used to evaluate the duration of in vivo persistence, function and trafficking of infused T cell clones. As a secondary objective, the residual measurable disease in these patients will also be followed to assess the therapeutic efficacy, if any, of these infusions. The estimated time to completion of the study is 24 months.

5. PATIENT ELIGIBILITY

5.1 Inclusion Criteria

5.1.1 Histopathological documentation of melanoma concurrent with the diagnosis of metastatic disease.

5.1.2 Male or female subject, 18 to 75 years of age

5.1.3 Expression of HLA-A2 or A3 as determined by FHCRC HLA typing lab.

5.1.4 Karnofsky Performance status of at least 80% (Appendix B) and an expected survival of greater than 6 months

5.1.5 Bi-dimensionally measurable disease by palpation on clinical exam, or radiographic imaging (X-ray, CT scan).

5.1.6 ANC >2,000/µLand platelet count >100,000/µL (at the time of first T cell infusion).

5.2 Exclusion Criteria

5.2.1 Pregnant women, nursing mothers, men or women of reproductive ability who are unwilling to use effective contraception or abstinence. Women of childbearing potential must have a negative pregnancy test within two weeks prior to entry.

5.2.2 Serum creatinine > 2.0 mg/dL or Creatinine clearance < 60 ml/min.

5.2.3 Significant hepatic dysfunction (SGOT > 150 IU or > 3x upper limit of normal; bilirubin > 1.6 mg/dL; prothrombin time > 1.5 x control).

5.2.4 Clinically significant pulmonary dysfunction, as determined by medical history and physical exam. Patients so identified will undergo pulmonary functions testing and those with FEV1 < 1.0 L or DLco (corr for Hgb) < 45% will be excluded.

5.2.5 Significant cardiovascular abnormalities as defined by any one of the following:

- congestive heart failure,
- clinically significant hypotension,
- symptoms of coronary artery disease,
- presence of cardiac arrhythmias on EKG requiring drug therapy

5.2.6 Central nervous system metastases at time of therapy. Patients with a prior history of CNS involvement but since treated, may be considered if no evidence of CNS disease is detected at least 2 months after treatment.

5.2.7 Patients with active infections or oral temperature > 38.2 C within 72 hours of study entry or systemic infection requiring chronic maintenance or suppressive therapy.

5.2.8 Chemotherapeutic agents (standard or experimental), radiation therapy, or other immunosuppressive therapies less than 3 weeks prior to T cell therapy. (Patients with bulky disease may undergo cytoreductive chemotherapy but treatment will be discontinued at least 3 weeks prior to T cell therapy).

5.2.9 Concurrent treatment with steroids

5.3 Virology testing (including HIV and Hepatitis B) will be performed within 6 months of the first T cell infusion. These will not be used as eligibility criteria but as a baseline result.

6. STUDY AGENTS

6.1 Antigen-specific CD8+ cytotoxic T cell clones Generation and characterization

All T cells administered will be CD8+ class I MHC-restricted antigen-specific T cell clones derived from the peripheral blood lymphocytes of metastatic melanoma patients.

Methods employed to generate and qualify CD8+ antigen-specific T cell clones for infusions are detailed in Appendix A. Briefly, T cell clones demonstrating antigen-specific cytolytic function will be selected for:

a. CD3+, 8+ surface phenotype
b. Class I MHC restricted lysis of antigen-expressing target cells
c. Mycoplasma, fungal and bacterial sterility

7. INVESTIGATIONAL PLAN

7.1 Leukapheresis

Patients will undergo leukapheresis to obtain peripheral blood mononuclear cells (PBMC's) for the generation of antigen-specific T cells in the lab. Patients ineligible for leukapheresis because of poor venous access or patients who elect not to undergo leukapheresis but still wish to remain on the study may undergo a maximum of four weekly phlebotomies to obtain PBMC's necessary to establish T cell cultures. On each of these blood draws, 50 – 100 cc of blood will be obtained.

7.2 Dose And Administration

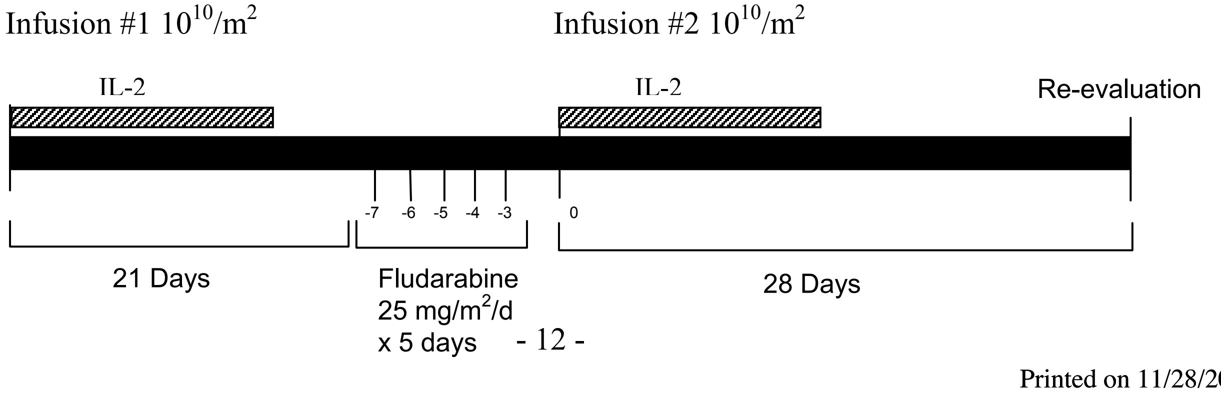

Antigen-specific CD8+ T cell clones will be administered at a dose of 10^{10} cells/m^2 for both Infusions #1 and #2. The median survival of transferred antigen-specific CTL clones in the absence of exogenous factors (e.g .IL-2) is less than 7 days. By the end of 14 days, the frequency of transferred CTL is < 0.1% of the total CD8+ population. Fludarabine 25 mg / m^2 will be infused daily for 5 days prior to the second infusion of antigen-specific CD8+ CTL clones (starting on Day –7). Four weeks after the second infusion, patients will be re-evaluated clinically and radiographically to determine the response to therapy.

7.2.1 T cell clones from in vitro cultures will be washed three times with RPMI-Hepes solution and resuspended in 250-500 ml of 0.9% NaCl in preparation for infusion.

7.2.2 T cells will be infused intravenously at a rate of not more than 500cc/hour through an 18 or 20 gauge catheter inserted into a peripheral vein or through a central catheter. Intravenous tubing will be provided for T cell administration and no filters will be used in the infusion line. The infusion bag will be gently mixed every 5 minutes during the infusion.

7.2.3 Subjects will have vital signs obtained by the Clinical Research Center (CRC) nurse preinfusion, every 15 minutes during the infusion, at the end of the infusion and hourly for 2 hours following the infusion.

7.2.4 Subjects will have oxygen saturation measured by pulse oximetry preinfusion, immediately post infusion and 2 hours post infusion.

7.2.5 Subjects will receive T cell infusions through the General Clinical Research Center (University of Washington).

7.3 Tumor Biopsy (Optional)

Patients undergoing surgery to remove or biopsy tumor as part of the usual management of their disease will be asked to consent to the use of a nondiagnostic portion of the tumor of the tumor for research purposes.
Patients may additionally be asked to consent to undergo a tumor biopsy for research purposes only should the tumor be accessible (e.g. skin or subcutaneous site) and risks in the opinion of the surgeon are minimal. New tumors may be biopsied when they appear following T cell infusion. Persistent tumors may be biopsied three weeks or longer following the last T cell infusion.

7.4 Management of Toxicities and Complications

7.4.1 Criteria for Removal of Patient from the Study

Toxicity grading will be evaluated according to guidelines in NCI Common Toxicity Criteria (66). If any patient develops grade 3 or higher toxicity related to the T cell therapy, no further infusions will be given to that patient and a regimen of corticosteroids will be given to patients.-

Exceptions:

The following toxicities are excluded as dose-limiting:

i. Fever > 40C for less than 24 hours
ii. WBC < 1000 for less than 4 weeks
iii. Platelets < 50,000 for less than 4 weeks
iv. ANC < 500 for less than 4 weeks
v. Lymphocytes < 500 for less than 4 weeks

7.4.2 Criteria for Discontinuation of Study

7.4.2.1 Dose-Limiting Toxicity (DLT)

If there exists sufficient evidence to suggest that the true DLT rate is in excess of 30%, the study will be terminated for safety reasons. (See Statistical Considerations, Section 12.2)

The study will be discontinued if

Dose-limiting toxicity (DLT) – (see section 7.4.4) is seen in

2 of the first 2 patients
3 of the first 5 or fewer patients
4 of the first 8 or fewer patients
5 of the first 10 or fewer patients
6 of the first 11 or fewer patients

7.4.2.2 'Rampant' or 'Rapidly Progressive' Disease

'Rampant' or 'Rapidly Progressive' Disease following immunosuppressive therapy has been described anecdotally in patients with metastatic melanoma undergoing nonmyeloablative allogeneic stem cell transplantation and is believed to be the result of severe and prolonged immunosupressive therapy. In contrast to patients receiving a single course of fludarabine, such patients received high-dose cyclophosphamide followed by prolonged immunosuppression with cyclosporine, MMF and/or steroid therapy. It is therefore unlikely that 'rampant' disease progression will be observed during the course of this study as a result of a single course of fludarabine. It is more likely that disease progression in patients on this study represents the natural history of refractory metastatic melanoma in which the average time to progression ranges from 2.4-4.9 months (67, 68). However, as a precautionary safeguard, interim analysis will be performed on the first 6 patients completing the study to Day 49. If

on Day 49 a three-fold increase in the sum of all measurable lesions (69) is observed compared to pre-treatment lesions in 4 or more of 6 patients, then the study will be suspended while the data is reviewed by an ad hoc Data Safety Monitoring Board consisting of at least two clinical investigators not associated with this study and a biostatistician. No additional patients will be treated until a decision is rendered by the Board as to whether the study should or should not continuee.

7.4.3. Management of Symptoms during Infusion

Mild transient symptoms have been observed with LAK and TIL cell infusions and, with infusions of antigen-specific T cell clones (protocol 1017). The management of these symptoms is outlined below.

a. Fever, chills and temperature elevations > 101°F will be managed with acetaminophen 650 mg p.o. q 4-6 hrs. All subjects that develop fever or chills will have a blood culture drawn.

b. Headaches may be managed with acetaminophen following a neurologic examination.

c. Nausea, vomiting will be treated with a non-steroidal anti-emetic of choice.

d. Hypotension will initially be managed by intravenous fluid administration and further measures as dictated by standard medical practice.

e. Hypoxemia will initially be managed with supplemental oxygen and further measures as dictated by standard medical practice.

7.4.4 Potential Toxicities Warranting Ablation of Adoptively Transferred Cytotoxic T Cell Clones

The following will be considered Dose-Limiting Toxicities (DLT):

a. Ophthalmologic: Patients receiving T cells targeting tyrosinase, MART-1 or gp100 will undergo a pre-therapy ophthalmologic exam and particular attention in the post-infusion monitoring period be given to ocular toxicity. The presence of retinitis or choroiditis as determined by ophthalmologic exam will be coded as a grade 3 toxicity and will warrant administration of steroids to ablate transferred T cells. If symptoms of visual field impairment arise, a formal visual fields examination will be conducted to determine if such a visual field defect exists. Any defect in the visual field will warrant T cell ablation.

b. Grade 2 or greater allergic reaction of: asymptomatic bronchospasm, or generalized urticaria

c. Grade 3 or greater toxicity (NCI Common Toxicity Criteria (66)) occurring in any other organ system following administration of antigen-specific cytotoxic

T cells and not attributable to underlying metastatic melanoma or other identifiable cause and excepting :

i. Fever > 40C for less than 24 hours
ii. WBC < 1000 for less than 4 weeks
iii. Platelets < 50,000 for less than 4 weeks
iv. ANC < 500 for less than 4 weeks
v. Lymphocytes < 500 for less than 4 weeks

7.4.5 Treatment with Corticosteroids

a. Patients will receive corticosteroids if treatment-related toxicity warranting ablation of T cells is observed

b. All patients will be hospitalized for the first 48 hours for monitoring. The following dose schedule will be used:

Day 1	Intravenous Solu-Medrol at 2 mg/kg
Day 2	Intravenous Solu-Medrol at 2 mg/kg
Day 3-4	Prednisone at 30 mg po b.i.d.
Day 5-6	Prednisone at 15 mg po b.i.d.
Day 7-8	Prednisone at 10 mg po b.i.d.
Day 9-10	Prednisone at 10 mg po q.d
Day11-12	Prednisone at 5 mg po q.d.

c. The in vivo frequency of infused CD4+ T cells will be assayed immediately prior to and 48 hours after the start of steroid therapy. If the frequency of infused CD4+ T cells as determined by peptide-tetramer and RT-PCR analysis of a clone-specific CDR3 region of the TCR at this time has not decrease to 25% or less of the T cell frequency prior to infusion, then, high-dose Solu-Medrol at 2 mg/kg will be given for an additional 48 hours before continuing with the tapering dose schedule.

7.4.6 Management of Chronic Toxicity

Based on animal models of T cell mediated autoimmunity, it is anticipated that if toxicity due to the infused T cells should occur, such events will be acute and, if significant, will result in the timely removal of the patient from the study and administration of steroids and/or discontinuation of the study. However, unforeseen chronic or delayed autoimmune toxicities may not permit a timely intervention. These patients will be treated high-dose steroids to mediate lympholytic and anti-inflammatory effects and any other measures to ameliorate toxicity, including chronic steroid therapy.

7.5 CONCOMITANT THERAPY

7.5.1 Active infections occurring after initiating the study should be treated according to the standard of care.

7.5.2 The following agents are not allowed while on study: systemic corticosteroids (except as outlined for management of toxicity of non-transduced CTL), immunotherapy (for example, interleukins, interferons, melanoma vaccines, intravenous immunoglobulin, expanded polyclonal TIL or LAK therapy), pentoxifylline, or other investigational agents.

7.6 PREMATURE DISCONTINUATION

Subjects who do not complete the study medication will be considered to have prematurely discontinued the study. The reasons for premature discontinuation (for example, voluntary withdrawal, toxicity, death) must be recorded on the case report form. A subject may re-enter the study after premature discontinuation only by approval of the Principal Investigator. If possible, final study evaluations should be completed at the time of discontinuation. Potential reasons for premature discontinuation include:

7.6.1 The development of a life-threatening infection.

7.6.2 Judgment of the principal investigator that the patient is too ill to continue.

7.6.3 Patient noncompliance with study therapy and/or clinic appointments.

7.6.4 Pregnancy.

7.6.5 Voluntary withdrawal; a patient may remove himself/herself from the study at any time without prejudice.

7.6.6 Significant and rapid progression of melanoma requiring alternative medical or surgical intervention including, but not limited to, the development of CNS metastasis.

7.6.7 Grade III or IV toxicity judged to be possibly or probably related to study therapy and fulfilling the stopping criteria as described in 7.4.2.

7.6.8 Termination of the study by the principal investigator, the Institutional Review Office or the Food and Drug Administration.

8. SCHEDULE OF EVALUATIONS

Please see Appendix D for Schedule

8.1 Immunologic Studies.

Patients receiving T cells will have 40 mls of heparinized blood and a 6ml gray top tube drawn immediately prior to each infusion and 24 hours after each infusion and once weekly for 10 weeks as outlined in appendix D. Samples will be used to evaluate the duration of in vivo persistence, function and trafficking of infused T cell clones. All blood samples will be kept at room temperature and sent to Rm D3-235, FHCRC

8.2 Efficacy Assessment

This will be applicable only for those patients who presented with measurable disease.

8.2.1 Clinical Response.

Radiographic imaging and clinical assessment of residual disease will be compared with pre-infusion assessment. A complete response will be defined as total regression of tumor, a partial response as 50% or more regression of the sum of the perpendicular diameters of all measurable disease, a minor response as less than 50% regression. This assessment will be performed at 4 and 8 weeks following the last infusion and then every 3 months until disease progression or intervening therapy.

8.2.2 Tumor immunophenotyping

In the absence of complete regression, immunophenotype analysis of the tumor pre-infusion will be compared with persistent tumor nodules or new tumor nodules post-infusion to evaluate for the outgrowth of antigen-loss tumor variants. New nodules will be sampled when they arise. Persistent disease will be sampled 2 weeks following the last T cell infusion. Immunophenotyping will evaluate the expression of targeted antigen (MART-1) as well as other melanoma antigens (e.g, gp100, tyrosinase) and will be performed by the Immunohistochemistry Laboratory at the University of Washington Medical Center. The level of expression will be scored by the pathologist in 'blinded' samples, as 0-4+ with a score of 0 being no expression, 1+, 25% of cells positive; 2+, 50% of cells, 3+, 75% of cells, 4+ 100% of cells. Antigen loss will be defined as the absence of expression of targeted antigens or a significant decrease in expression (defined as a ≥ 2 level decrease in score) in post-infusion sample as compared with the pre-infusion sample.. Persistent expression of non-targeted antigen, e.g. gp100, in the post-infusion tumor sample in the presence of antigen-loss as defined above will be considered evidence of outgrowth of antigen-loss variants as a result of immune targeting. Single cell suspensions of tumor samples will also be stained with anti-Class I and Class II antibody to evaluate HLA allele loss as a possible alternative mechanism of immune evasion.

8.2.3 T cell localization to disease sites

Localization of transferred antigen-specific CTL to tumor sites will be evaluated in selected patients with surgically accessible disease (disease that is accessible by needle or core biopsy, or that can be excised). Tumor sampling will be performed from 3 to 7 days following the most recent T cell infusion. Single cell suspensions will be prepared and stained to identify antigen-specific CTL by tetramer analysis. Tetramer positive cells will be sorted and analyzed for identity to the original infused CTL clone(s) by PCR of a clone-specific CDR3 region of the T cell receptor. Where possible, these results will be compared with a pre-infusion tumor sample and the peripheral blood. A T cell frequency at least five-fold higher than that found in the peripheral blood or pre-infusion sample will be considered evidence of T cell localization/accumulation in disease sites.

9. SAFETY ASSESSMENT (RELEVANT DATA BEING OBTAINED)

Physical exam and Blood Chemistry/Hematology results

Toxicities will be graded according to the Common Toxicity Criteria(66)

10. EFFICACY ASSESSMENT (RELEVANT DATA BEING OBTAINED)

- Radiographic imaging and clinical assessment of residual disease defined previously in post-surgical evaluation. A complete response will be defined as total regression of tumor, a partial response as 50% or more regression of the sum of the perpendicular diameters of all measurable disease, a minor response as less than 50% regression. This assessment will be performed on 4 and 8 weeks after the last infusion and then every 3 months thereafter until disease progression or intervening therapy.

- In vivo persistence of transferred antigen-specific CTL clones as measured by 1) direct assay of PBL for retroviral vector DNA by PCR 2) specific peptide-MHC tetramer staining of peripheral blood lymphocytes followed by flow cytometry analysis, 3) ELISPOT determination of cytokine release following peptide stimulation of peripheral blood lymphocytes and 4) generation of antigen-specific CTL. Isolation of such CTL from patients will demonstrate whether or not transferred CTL will survive and retain their lytic phenotype in vivo. The peak frequency and median survival of T cells following infusions without and with fludarabine conditioning will be determined.

11. REPORTING ADVERSE EVENTS

All unexpected and serious adverse events which may be due to study treatment or intervention must be reported to the FHCRC Institutional Review Office as soon as possible but within at least 7 calendar days of the investigator learning of the event.

11.1 Definition of an Adverse Event

Any untoward medical occurrence in a patient or clinical investigation subject administered a pharmaceutical product, medical treatment or procedure and which does not necessarily have to have a causal relationship with this treatment.

1. **Life-threatening Adverse Event**
   Any adverse event that places the patient or subject, in view of the investigator, at immediate risk of death from the reaction.

2. **Unexpected Adverse Event**
   An unexpected adverse event is an adverse event that is not described in the study protocol or informed consent.

3. **Serious Adverse Event (SAE)**
   Any adverse event occurring that results in any of the following outcomes:

- death,
- a life-threatening adverse event (real risk of dying),
- inpatient hospitalization or prolongation of existing hospitalization,
- a persistent or significant disability/incapacity,
- a congenital anomaly
- requires intervention to prevent permanent impairment of damage.

11.2 Attribution -

- Related – includes adverse events that are definitely, probably, or possibly related to the medical treatment or procedure.
- Not Related – includes adverse events are doubtfully related or clearly not related to the medical treatment or procedure.

11.3 Procedure for Reporting Adverse Events

The FHCRC Serious Adverse Event (SAE) Report Form will be completed for all adverse events that meet the expedited reporting requirements. The SAE form will be faxed to the IRO at (206) 667-6831. All available information should be submitted but it is acceptable to fax an incomplete report form at the initial report. A completed report should be faxed as soon as possible but must be received within 15 calendar days.

Serious adverse events that do not meet the requirement for expedited reporting (not related to study treatment or expected) must be reported to the IRB as part of the annual renewal of the protocol.

12. STATISTICAL CONSIDERATIONS

12.1. Sample Size

The sample size of this clinical trials is modest but sufficiently powerful to fulfill the primary goal of this proposal – determine if prior lymphodepletion extends the survival

of transferred antigen-specific CD8+ T cell clones. The major immunologic endpoint, in vivo survival of transferred T cells was used to determine patient sample size.

For each patient, the T cell survival of infused CD8+ CTL given alone (Infusion #1) will be compared with subsequent infusion (Infusion #2) given with prior fludarabine conditioning. Since the second infusion given occurs 2 weeks after the first infusion at a time when transferred CD8 cells have disappeared from the circulation, residual cells from the first infusion will not be a confounding factor since, in our previous study, transferred CD8+ T cells were undetectable at two weeks.

Statistical modeling indicates that, with a sample size of 12 patients, and assumed true difference in T cell survival with fludarabine conditioning compared to infusions without of at least 4 days with a standard deviation (SD) of 2 days, we shall have approximately 99% power to detect a statistically significant difference in T cell survival at the two-sided level of significance of .05 (one-sample t-test). These power estimates do not depend on the actual assumed-true values, rather only on the difference between the values. The design of this trial using the first infusion of CD8 T cells administered alone as a baseline for each patient permits intra-patient analysis using paired samples with increased statistical power.

12.2 Dose-Limiting Toxicity

If there exists sufficient evidence to suggest that the true DLT rate is in excess of 30%, the study will be terminated for safety reasons. Sufficient evidence will be taken to mean any observed proportion of DLT's with a lower bound to the associated one-sided 80% confidence interval that exceeds 30%. Operationally, the following observed proportions would lead to such a bound: 2 of the first 2 patients, 3 of the first 5 or fewer, 4 of the first 8 or fewer, 5 of the first 10 or fewer, or 6 of the first 11 patients experience a DLT.

If the true DLT rate is 20%, the probability of stopping after 10 patients is approximately .15. If the true DLT rate is 50%, the probability of stopping after 10 patients is approximately .73 (estimated from Monte Carlo simulations).

**TARGETED/PLANNED ENROLLMENT:**<br>**Number of Subjects (*must provide actual numbers. I.e. no range)***			
**ETHNIC CATEGORY**	***Sex/Gender***		
	Females	Males	Total
Hispanic or Latino			
Not Hispanic or Latino	6	6	12
Ethnic Category Total of All Subjects*	6	6	12
**Racial Categories**			
American Indian/Alaska Native			
Asian			
Native Hawaiian or Other Pacific Islander			
Black or African American			
White			
Racial Categories: Total of All Subjects *	6	6	12

Neither gender nor ethnicity are criteria for enrollment on this study. University of Washington is a major referral center for patients with melanoma in the Northwest. Based on our previous enrollment experience with patients with metastatic melanoma who are HLA-A2+, the gender and ethnicity expected is shown below. Since melanoma afflicts primarily (> 95%) a Caucasian population, we do not expect to see significant minority representation. :

13. ADMINISTRATIVE CONSIDERATIONS

13.1 Institutional Review Board

In accordance with federal regulations (21 CFR 312.66), an Institutional Review Board (IRB) that complies with regulations in 21 CFR 56 must review and approve this protocol and the informed consent form prior to initiation of the study.

13.2 CONSENT

The Principal Investigator or his associate must explain verbally and in writing the nature, duration, and purpose of the study and possible consequences of treatment. Patients must also be informed that they may withdraw from the study at any time and for any reason without jeopardizing their future treatment. In accordance with federal regulations (21 CFR 312), all patients must sign the IRB-approved consent form in the presence of a witness. Prior to the start of the study, a copy of the IRB-approved consent form must be submitted to the Sponsor.

13.3 TERMINATION OF STUDY

The Principal Investigators reserve the right to terminate this study at any time. The FDA may also terminate the study.

APPENDIX A

I. Generation of CD8+ Antigen-specific T cell clones

Peripheral blood mononuclear cells (PBL) are obtained from the patient with melanoma by leukapheresis. The advantages to the leukapheresis procedure are: a one-time commitment of 4 hours obviating the need for repeated visits to have blood drawn, the approximately 20-fold greater yield of PBL compared to repeated blood draws, thus enabling us to set up many more T cell cultures simultaneously and increasing the probability of obtaining antigen-specific T cell clones from each patient. The PBL are washed twice in sterile phosphate buffered saline (PBS) supplemented with 1.488 g/L of EDTA (Invitrogen Corp.), and once in PBS.PBL are frozen at no more than 2x 0^8 cells/ml in freezing medium consisting of RPMI, 25mM L-glutamine, 30% human AB human serum and 10% DMSO (Sigma).

PBL are thawed and re-suspended in AIM-V medium at 3x10^8 cells/ml, and then placed in sterile tissue culture dishes at 3mL/well for separation into adherent and non-adherent populations by culture for 2 hours at 37º C. The adherent cells are treated with GM-CSF (Endogen) and IL-4 (Endogen) for 6 days at which time, the PBL-derived dendritic cells (DC) growing in the culture are matured for 2 days using autologous monocyte conditioned medium. Excess DC are frozen to be used for 2nd and 3rd stimulations.
1.8x10^6 DC are pulsed with 40 ucg/mL of GMP-certified peptide and 3ucg/mL of beta-2 microglobulin at a cell concentration of 2x10^6/mL in PBS with 1% HSA. The DC are gamma-irradiated (5000 rads), washed, and re-suspended in medium containing RPMI, 25mM HEPES, 2mM L-glutamine, and 10% human AB serum (CTLmedium). DC will then be added to 70x10^6 PBL and plated in 48-well tissue culture treated plates at 1mL/well. Cultures will then be incubated for seven days at 37ºC in 5.0% CO2 in a Forma 3950 incubator. The cultures are re-stimulated twice with gamma-irradiated stimulator cells (thawed DC or monocytes) and pulsed with 10ucg/mL of peptide and 3ucg/mL of beta-2 microglobulin..

After a total of three stimulations, all cell lines are tested in a chromium release assay for lytic activity against peptide-pulsed and unpulsed HLA-matched targets, antigen-positive and antigen-negative tumor targets prior to T cell cloning to confirm the presence of antigen-specific cytolytic activity. T cells are plated at limiting dilution in 96-well round-bottomed plates. Each well will receive 30ng/mL of anti-CD3 (OKT3, ORTHOCLONE), $7.5x10^4$ gamma irradiated (4500 rads) allogeneic PBL and $1x10^4$ gamma irradiated (8000 rads) allogeneic LCL as feeder cells in 0.2 mL of culture media containing 50 units IL-2 per mL. Cryopreserved allogeneic for feeder cells are obtained by leukapheresis of donors screened by the standard tested for donated blood at the Puget Sound Blood Center. Allogeneic LCL (or EBV-transformed lymphoblastoid cell lines) are derived from the TM-LCL line which has been approved by the FDA for use in adoptive therapy..

Wells positive for growth are identified 10 to 14 days after plating and are screening in a microcytotoxicity assay to identify those clones with Class I MHC-restrictricted cytolytic activity for antigen-expressing target cells. Positive clones are re-stimulated and expanded in CTL medium in 25cm2 flasks with anti-CD3 monoclonal antibody and irradiated allogeneic PBL and LCL added as feeder cells. The cultures are fed with IL-2 50 U/mL 24 hours after re-stimulation and every 48 to 96 hours thereafter. All clones are retested for Class I MHC restricted anti-specific cytolytic activity and tested for cell surface phenotype (CD3, CD4 and CD8). T cell clones demonstrating rapid in vitro growth, Class I MHC restricted antigen-specific cytolytic activity and a $CD3^+$,$CD8^+$ and $CD4^-$ phenotype are selected for a second expansion in CTL medium using fetal bovine serum instead of human AB serum. All clones are retested for Class I MHC restricted antigen-specific cytolytic activity.

All clones derived from a given study patient are labeled with the patient's initials followed by the name of the peptide for which they are specific and a number designating that specific clone. Data on cell growth and characteristics for each clone are separately recorded and these records retained. Each recipient has a separate laboratory record book for records on growth and characteristics of the T cell clones derived and grown from that individual. All records with the results of testing performed on the T cell clones are entered into the record book.

When the GMP cell sorting facility becomes available (expected date: July 2003), the following approach may be adopted for the generation of T cell clones.

A dendritic cell-enriched population of adherent PBMCs are harvested following 7-day incubation with GM-CSF and IL-4. They are pulsed with the peptide of interest and co-cultivated $CD4^-$ depleted responder T cells in 6-well plates. IL-2 (10 U/mL) is added the following day. After 7 days in culture, the cultures are harvested, washed and re-suspended in staining solution (1% human serum in PBS) containing 50 ucg/mL of PE-conjugated specific peptide-tetramer and anti-CD8 FITC antibody (Caltag) at a cell concentration of 10^7 cells/mL.After one hour at 4C°, the cells are washed and re-suspended in staining solution for flow cytometry analysis and cell sorting. $CD8^+$ $tetramer^+$ cells are analyzed and sorted under GMP conditions using the high-speed cell sorter at the University of Washington Clinical Research Center facility. They are directly cloned into 96-well plates containing irradiated feeder cells ($5x10^4$ PBL/well and 10^4 LCL/well) with 30 ng/mL of a CD3 and IL-2 (50 U/mL). Wells positive for clonal growth are selected 10 to 14 days after plating and screened in a microcytotoxicity assay to identify those clones with cytolytic activity for antigen+tumor targets. The use of tetramers is expected to decrease the time from patient enrollment (leukapheresis) to T cell infusion from 2 1/2 months to 6 weeks.

Table 1. Candidate Target Antigens, HLA restriction and Epitope sequence

Antigen	HLA	A.A.	Peptide Sequence
TYROSINASE	***A2***	368-377	YMDGTMSQV
GP100	**A2**	154-162	KTWGQYWQV
MART1	**A2**	26-35	EAAGIGILTV
MAGE-1	**A3**	96-104	SLFRAVITK
MAGE-4	**A2**	230-239	GVYDGREHTV
MAGE-10	**A2**	254-262	GLYDGMEHL
NY-ESO-1	**A2**	157-170	SLLMWITQCFLPVF

II. Characterization of Antigen-specific CD8 T cell Clone Prior to Use in Adoptive Immunotherapy

Table 2. Validation Testing of T cell clones

Test	Result
Chromium Release Assay	Lysis > 25%
Class I MHC Restriction	>75% Decreased lysis when using Class I-mismatched targets.
FACS analysis of surface antigens	CD3+, CD4-, CD8+
Mycoplasma-Genprobe	Negative
Sterility	No bacterial, fungal growth

APPENDIX B

Karnofsky Performance Status Scale

	%	
Able to carry on normal activity; no special care is needed	100	Normal; no complaints; no evidence of disease
	90	Able to carry on normal activity; minor signs or symptoms of disease
	80	Normal activity with effort; some signs or symptoms of disease
Unable to work; able to live at home; cares for most personal needs; a varying amount of assistance is needed	70	Cares for self; unable to carry on normal activity or do active work
	60	Requires occasional assistance by is able to care for most needs
	50	Requires considerable assistance and frequent medical care
Unable to care for self; requires equivalent of institutional or hospital care; disease may be progressing rapidly	40	Disabled, requires special care and assistance
	30	Severely disabled; hospitalization is indicated through death not imminent
	20	Very sick; hospitalization is necessary
	10	Moribund; fatal processes progressing rapidly
	0	DEAD

From Karnofsky et al. Cancer 1948; 1:634-669

APPENDIX C

DATA AND SAFETY MONITORING PLANS FOR PROTOCOL 1796

Phase I Study To Evaluate The Safety Of Cellular Adoptive Immunotherapy Using Autologous CD8+ Antigen-Specific T Cell Clones Following Fludarabine Lymphodepletion For Patients With Metastatic Melanoma

REPORTING ADVERSE EVENTS

Definition of an Adverse Event

Any sign , symptom or illness that appears to worsen during the study period regardless of the relationship to the study agent is an adverse event.

Obligation of Investigator

All adverse events occurring during the study, whether or not attributed to the study agent, that are observed by the Investigator or reported by the patient will be recorded on the Case Report Form. The investigator will be responsible for reporting adverse events to the IRB, FDA and NIH. Attributes will include a description, onset and resolution date, duration, maximum severity, assessment of relationship to the study agent or other suspect agent(s), action taken and outcome.

Adverse events will be graded accordingly: 0 = none, 1 = mild, 2 = moderate, 3 = severe, 4 = life-threatening or debilitating, and 5 = fatal. Association or relatedness to the study agent will be graded as follows: 1 = unrelated, 2 = unlikely, 3 = possibly, 4 = probably, and 5 = definitely related.

Serious adverse events

Serious adverse events occurring during or after completion of therapy are defined as any one of the following:

1. patient death, regardless of cause, occurring within 30 days of study agent administration
2 life-threatening event
3. prolonged hospitalization or requirement for additional hospitalizations during treatment and monitoring period
4. persistent or significant disability or incapacity

A life-threatening event is defined as having placed the patient, in the view of the Investigator, at immediate risk of death from the adverse event as it occurred. It does not include an adverse event that, had it occurred in a more serious form, might have caused death.

All adverse events that do not meet at least one of the above criteria are defined as non-serious.

Assessment of the cause of the event has no bearing on the assessment of the event's severity.

Unexpected adverse events

Unexpected adverse events are those which are symptomatically and pathophysiologically related to a known toxicity but differ because of greater severity or specificity.

Procedure for Reporting Adverse Events

For all serious adverse events that are unexpected and related to treatment, a telephone report followed by a detailed written report will be submitted within 3 working days to the FDA and IRB. All other deaths and unexpected serious adverse events will be reported at the time of annual renewal. The report must include the date and time of onset, severity and duration of the event, the relationship to study drug, the treatment given, and the eventual outcome.

Appropriate clinical, diagnostic, and laboratory measures to attempt to delineate the cause of the adverse reaction in question must be performed and the results reported. All tests that reveal an abnormality considered to be drug-related will be repeated at appropriate intervals until the course is determined or a return to normal values occurs.

All adverse events will be recorded on case report forms provided by the Investigator. Information will be recorded as noted above.

DATA AND SAFETY MONITORING

The data and safety monitoring plans for this trial have been discussed an approved by the institutional Clinical Investigators Meeting which provides an open forum for presentation and discussion of the clinical protocol and the Scientific Review Committee (SRC) which provides formal internal peer review of all protocols and general scientific oversight of clinical research.

Obligation of Investigator

All data and safety issues will be monitored by the Investigator with the assistance of the protocol Research Coordinator and Research Technicians conducting the immunobiologic assays related to the study.

Safety Considerations

Safety considerations for this Phase I trial include

1. Definition for Dose-limiting toxicities
1. Stopping Criteria

Patients are monitored at prescribed regular intervals during and following T cell administration as described in the clinical protocol. Relevant data being obtained from safety assessments include

- Physical exam and Blood Chemistry/Hematology results; Neurologic exam and Ophthalmologic exam
- Adverse event reporting

Definition for Dose-limiting Toxicities

Serious toxicity is defined as Grade 3 or greater toxicity according to the Common Toxicity Scale (Appendix C of protocol).

Stopping Criteria

If any patient develops grade 3 or higher toxicity related to the T cell therapy, no further infusions will be given to that patient and a regimen of corticosteroids will be given to patients.-

Exceptions:

The following toxicities are excluded as dose-limiting:

vi. Fever > 40C for less than 24 hours
vii. WBC < 1000 for less than 4 weeks
viii. Platelets < 50,000 for less than 4 weeks
ix. ANC < 500 for less than 4 weeks
x. Lymphocytes < 500 for less than 4 weeks
xi.

Criteria for Discontinuation of Study

The study will be discontinued if

Dose-limiting toxicity (DLT) – (see section 7.4.4) is seen in

2 of the first 2 patients
3 of the first 5 or fewer patients
4 of the first 8 or fewer patients
5 of the first 10 or fewer patients
6 of the first 11 or fewer patients

'Rampant' or 'Rapidly Progressive' Disease

'Rampant' or 'Rapidly Progressive' Disease following immunosuppressive therapy has been described anecdotally in patients with metastatic melanoma undergoing nonmyeloablative allogeneic stem cell transplantation and is believed to be the result of severe and prolonged immunosupressive therapy. In contrast to patients receiving a single course of fludarabine, such patients received high-dose cyclophosphamide followed by prolonged immunosuppression with cyclosporine, MMF and/or steroid therapy. It is therefore unlikely that 'rampant' disease progression will be observed during the course of this study as a result of a single course of fludarabine. It is more likely that disease progression in patients on this study represents the natural history of refractory metastatic melanoma in which the average time to progression ranges from 2.4-4.9 months (67, 68). However, as a precautionary safeguard, interim analysis will be performed on the first 6 patients completing the study to Day 49. If on Day 49 a three-fold increase in the sum of all measurable lesions (69) is observed compared to pre-treatment lesions in 4 or more of 6 patients, then the study will be suspended while the data is reviewed by an ad hoc Data Safety Monitoring Board consisting of at least two clinical investigators not associated with this study and a biostatistician. No additional patients will be treated until a decision is rendered by the Board as to whether the study should or should not continuee.

Premature Discontinuation

Subjects who do not complete the study medication will be considered to have prematurely discontinued the study. The reasons for premature discontinuation (for example, voluntary withdrawal, toxicity, death) must be recorded on the case report form. A subject may re-enter the study after premature discontinuation only by approval of the Principal Investigator. If possible, final study evaluations should be completed at the time of discontinuation. Potential reasons for premature discontinuation include:

1. The development of a life-threatening infection.
2. Judgment of the principal investigator that the patient is too ill to continue.
3. Patient noncompliance with study therapy and/or clinic appointments.
4. Pregnancy.
5. Voluntary withdrawal; a patient may remove himself/herself from the study at any time without prejudice.
6. Significant and rapid progression of melanoma requiring alternative medical or surgical intervention including, but not limited to, the development of CNS metastasis.
7. Grade III or IV toxicity judged to be possibly or probably related to study therapy.
8. Termination of the study by the principal investigator, or the Food and Drug Administration.

ENROLLMENT RATE RELATIVE TO EXPECTATION, PATIENT CHARACTERISTICS, COMPETING PROTOCOLS

The Melanoma Clinic at the Univ of Washington sees, in addition to its registered pool of melanoma patients, over 115 new melanoma patients per year and is the major referral site for patients in the Northwest states of Washington, Alaska, Montana and Idaho. Approximately 44% (n=49) of these new patients from 9/1/98 to 8/31/99 had stage IV disease. In addition, an average of 75 patients with stage IV disease are being seen on a regular basis in the melanoma clinic. (Total number of patients being seen with stage IV disease : 124. This does not include patients with Stage III or earlier disease who progress to Stage IV disease and become eligible for this protocol). Patients enrolled will be 18-75 years of age (98% of patients, n=122) with Karnofsky's performance status of > 70% (85% of these patients, n=104) and HLA-A2+ (40% of these patients, n=42). Of these 42 patients, approximately two-thirds present with surgically accessible disease (n=28). Patients will enroll on this protocol as first-line therapy or for therapy of refractory disease. A fraction of patients with refractory disease are also eligible for treatment with a nonmyeloablative marrow transplant if a matched sibling is found. The probability of a matched sibling in any household, given an average family size of 3.2 (82) is <30 %. Attrition of HLA-A2+ patients to this transplant protocol will be < 6 /year. The remaining pool of over 20 patients/year exceeds our recruitment requirement of 10 patients/year. Our center has given this protocol first priority for Stage IV melanoma patients and based on the ongoing accrual rate, we anticipate that the enrollment for the targeted accrual of 20 patients over two years can be completed in 18-20 months.

APPENDIX D

		Vital Signs O2 Saturation	Other	Blood Draw				Staging for measurable dis.
				Comprehensive Panel**	CBC diff, plts	Research Green	Bld Gray	
**INFUSION #1**	**Day 0 Pre-Infusion**	x	IV placement	x	x	x	x	x
	**During Infusion Q 15 min. x 1 hr**	x	T cell infusion					
	**Post-Infusion Q 30 min. x 2 hr**	x						
	**VS Q6H**	x	Overnight Stay					
	**Day +1**	x	IV removal Discharge home	x	x	x	x	
	**Day +3**			x	x	x		
	**Day +7**	x		x	x	x	x	
	**Day+ 14**	x		x	x	x	x	
Pre-infusion Fludarabine	**Day +21**	x	IV placement Administer FLU	x	x	x	x	
Pre-infusion Fludarabine	Day +22	x	IV placement Administer FLU		x			
Pre-infusion Fludarabine	Day +23	x	IV placement Administer FLU		x		x	
Pre-infusion Fludarabine	Day +24	x	IV placement Administer FLU		x			
Pre-infusion Fludarabine	Day +25	x	IV placement Administer FLU		x		x	
	Day +26				x			
	Day +27				x			
**INFUSION #2**	**Day+ 28 Pre-Infusion**	x	IV placement	x	x	x	x	
	**During Infusion Q 15 min. x 1 hr**	x	T cell infusion					
	**Post-Infusion Q 30 min. x 2 hr**	x						
	**VS Q6H**	x	Overnight Stay					
	**Day +29**	x	IV removal Discharge home	x	x	x	x	
	Day+31	x		x	x	x		
	Day+35	x		x	x	x	x	
	Day+42	x		x	x	x	x	
	Day+49	x		x	x	x	x	
	Day +56	x		x	x	x	x	X (D+56 to D +70)

** includes electrolytes, BUN, Cr, Liver function tests, LDH

*** 40 ml in heparinzied tube and 5-10ml gray top to D3-220, Thomas Bldg, FHCRC

compare with pre-infusion staging exam

LITERATURE CITED

1. DeVita, V.T., S. Hellman, and S.A. Rosenberg. 2000. Cancer Principles & Practice of Oncology. J. B. Lippincott Co., Philadelphia.
2. Greenlee, R.T., M.B. Hill-Harmon, T. Murray, and M. Thun. 2001. Cancer Statistics, 2001. *CA: A Cancer Journal for Clinicans* 51:15-36.
3. Mazumder, A., T.J. Eberlein, E.A. Grimm, D.J. Wilson, A.M. Keenan, R. Aamodt, and S.A. Rosenberg. 1984. Phase I study of the adoptive immunotherapy of human cancer with lectin activated autologous mononuclear cells. *Cancer* 53:896-905.
4. Grimm, E.A., A. Mazumder, H.Z. Zhang, and S.A. Rosenberg. 1982. Lymphokine-activated killer cell phenomenon. Lysis of natural killer-resistant fresh solid tumor cells by interleukin 2-activated autologous human peripheral blood lymphocytes. *Journal of Experimental Medicine* 155:1823-1841.
5. Rosenberg, S.A., M.T. Lotze, L.M. Muul, S. Leitman, A.E. Chang, S.E. Ettinghausen, Y.L. Matory, J.M. Skibber, E. Shiloni, J.T. Vetto, and et al. 1985. Observations on the systemic administration of autologous lymphokine-activated killer cells and recombinant interleukin-2 to patients with metastatic cancer. *N Engl J Med* 313:1485-1492.
6. Rosenberg, S.A., M.T. Lotze, L.M. Muul, A.E. Chang, F.P. Avis, S. Leitman, W.M. Linehan, C.N. Robertson, R.E. Lee, and J.T. Rubin. 1987. A progress report on the treatment of 157 patients with advanced cancer using lymphokine-activated killer cells and interleukin-2 or high-dose interleukin-2 alone. *New England Journal of Medicine* 316:889-897.
7. Rosenberg, S.A., P. Spiess, and R. Lafreniere. 1986. A new approach to the adoptive immunotherapy of cancer with tumor-infiltrating lymphocytes. *Science* 233:1318-1321.
8. Itoh, K., A.B. Tilden, and C.M. Balch. 1986. Interleukin 2 activation of cytotoxic T-lymphocytes infiltrating into human metastatic melanomas. *Cancer Research* 46:3011-3017.
9. Kradin, R.L., L.A. Boyle, F.I. Preffer, R.J. Callahan, M. Barlai-Kovach, H.W. Strauss, S. Dubinett, and J.T. Kurnick. 1987. Tumor-derived interleukin-2-dependent lymphocytes in adoptive immunotherapy of lung cancer. *Cancer Immunology, Immunotherapy* 24:76-85.
10. Topalian, S.L., L.M. Muul, D. Solomon, and S.A. Rosenberg. 1987. Expansion of human tumor infiltrating lymphocytes for use in immunotherapy trials. *Journal of Immunological Methods* 102:127-141.
11. Rosenberg, S.A., J.R. Yannelli, J.C. Yang, S.L. Topalian, D.J. Schwartzentruber, J.S. Weber, D.R. Parkinson, C.A. Seipp, J.H. Einhorn, and D.E. White. 1994. Treatment of patients with metastatic melanoma with autologous tumor-infiltrating lymphocytes and interleukin 2 [see comments]. *Journal of the National Cancer Institute* 86:1159-1166.
12. Topalian, S.L., D. Solomon, F.P. Avis, A.E. Chang, D.L. Freerksen, W.M. Linehan, M.T. Lotze, C.N. Robertson, C.A. Seipp, and P. Simon. 1988. Immunotherapy of patients with advanced cancer using tumor-infiltrating lymphocytes and recombinant interleukin-2: a pilot study. *Journal of Clinical Oncology* 6:839-853.

13. Greenberg, P.D. 1991. Adoptive T cell therapy of tumors: mechanisms operative in the recognition and elimination of tumor cells. *Advances in Immunology* 49:281-355.
14. van der Bruggen, P., C. Traversari, P. Chomez, C. Lurquin, E. De Plaen, B. Van den Eynde, A. Knuth, and T. Boon. 1991. A gene encoding an antigen recognized by cytolytic T lymphocytes on a human melanoma. *Science* 254:1643-1647.
15. Vandeneynde, B.J., and T. Boon. 1997. TUMOR ANTIGENS RECOGNIZED BY T LYMPHOCYTES [Review]. *International Journal of Clinical & Laboratory Research* 27:81-86.
16. Coulie, P.G., V. Brichard, A. Van Pel, T. Wolfel, J. Schneider, C. Traversari, S. Mattei, E. De Plaen, C. Lurquin, and J.P. Szikora. 1994. A new gene coding for a differentiation antigen recognized by autologous cytolytic T lymphocytes on HLA-A2 melanomas [see comments]. *Journal of Experimental Medicine* 180:35-42.
17. Brichard, V., A. Van Pel, T. Wolfel, C. Wolfel, E. De Plaen, B. Lethe, P. Coulie, and T. Boon. 1993. The tyrosinase gene codes for an antigen recognized by autologous cytolytic T lymphocytes on HLA-A2 melanomas. *Journal of Experimental Medicine* 178:489-495.
18. Van den Eynde, B.J., and P. van der Bruggen. 1997. T cell defined tumor antigens. *Current Opinion in Immunology* 9:684-693.
19. Ohman Forslund, K., and K. Nordqvist. 2001. The melanoma antigen genes--any clues to their functions in normal tissues? *Experimental Cell Research* 265:185-194.
20. Kawakami, Y., S. Eliyahu, C.H. Delgado, P.F. Robbins, L. Rivoltini, S.L. Topalian, T. Miki, and S.A. Rosenberg. 1994. Cloning of the gene coding for a shared human melanoma antigen recognized by autologous T cells infiltrating into tumor. *Proceedings of the National Academy of Sciences of the United States of America* 91:3515-3519.
21. Kawakami, Y., and S.A. Rosenberg. 1996. T-cell recognition of self peptides as tumor rejection antigens. *Immunol Res* 15:179-190.
22. Houghton, A.N., S. Vijayasaradhi, B. Bouchard, C. Naftzger, I. Hara, and P.B. Chapman. 1993. Recognition of autoantigens by patients with melanoma. *Annals of the New York Academy of Sciences* 690:59-68.
23. Riddell, S.R., and P.D. Greenberg. 1990. The use of anti-CD3 and anti-CD28 monoclonal antibodies to clone and expand human antigen-specific T cells. *Journal of Immunological Methods* 128:189-201.
24. Yee, C., P.A. Savage, P.P. Lee, M.M. Davis, and P.D. Greenberg. 1999. Isolation of high avidity melanoma-reactive CTL from heterogeneous populations using peptide-MHC tetramers. *Journal of Immunology* 162:2227-2234.
25. Yee, C., M.M. Davis, and P.P. Lee. 2000. Methods for Use of Peptide-MHC Tetramers in Tumor Immunology. *In* Methods in Molecular Medicine – Melanoma. B. Nickoloff, editor. Academic Press. 353-362.
26. Yee, C., J.A. Thompson, P. Roche, D.R. Byrd, P.P. Lee, M. Piepkorn, K. Kenyon, M.M. Davis, S.R. Riddell, and P.D. Greenberg. 2000. Melanocyte destruction after antigen-specific immunotherapy of melanoma: direct evidence of t cell-mediated vitiligo. *J Exp Med* 192:1637-1644.

27. Yee, C., J.A. Thompson, D. Byrd, S.R. Riddell, P. Roche, E. Celis, and P.D. Greenberg. 2002. Adoptive T cell therapy using antigen-specific CD8+ T cell clones for the treatment of patients with metastatic melanoma: In vivo persistence, migration, and antitumor effect of transferred T cells. *Proc Natl Acad Sci U S A* 99:16168-16173.
28. Jiang, L.Q., M. Jorquera, and J.W. Streilein. 1993. Subretinal space and vitreous cavity as immunologically privileged sites for retinal allografts. *Investigative Ophthalmology & Visual Science* 34:3347-3354.
29. Taylor, A.W., J.W. Streilein, and S.W. Cousins. 1992. Identification of alpha-melanocyte stimulating hormone as a potential immunosuppressive factor in aqueous humor. *Current Eye Research* 11:1199-1206.
30. Taylor, A.W., and J.W. Streilein. 1996. Inhibition of antigen-stimulated effector T cells by human cerebrospinal fluid. *Neuroimmunomodulation* 3:112-118.
31. Bystryn, J.C., D. Rigel, R.J. Friedman, and A. Kopf. 1987. Prognostic significance of hypopigmentation in malignant melanoma. *Archives of Dermatology* 123:1053-1055.
32. Rosenberg, S.A., and D.E. White. 1996. Vitiligo in patients with melanoma: normal tissue antigens can be targets for cancer immunotherapy. *Journal of Immunotherapy with Emphasis on Tumor Immunology* 19:81-84.
33. Reusser, P., S.R. Riddell, J.D. Meyers, and P.D. Greenberg. 1991. Cytotoxic T-lymphocyte response to cytomegalovirus after human allogeneic bone marrow transplantation: pattern of recovery and correlation with cytomegalovirus infection and disease. *Blood* 78:1373-1380.
34. Li, C.R., P.D. Greenberg, M.J. Gilbert, J.M. Goodrich, and S.R. Riddell. 1994. Recovery of HLA-restricted cytomegalovirus (CMV)-specific T-cell responses after allogeneic bone marrow transplant: correlation with CMV disease and effect of ganciclovir prophylaxis. *Blood* 83:1971-1979.
35. Walter, E.A., P.D. Greenberg, M.J. Gilbert, R.J. Finch, K.S. Watanabe, E.D. Thomas, and S.R. Riddell. 1995. Reconstitution of cellular immunity against cytomegalovirus in recipients of allogeneic bone marrow by transfer of T-cell clones from the donor [see comments]. *New England Journal of Medicine* 333:1038-1044.
36. Lenardo, M., K.M. Chan, F. Hornung, H. McFarland, R. Siegel, J. Wang, and L. Zheng. 1999. Mature T lymphocyte apoptosis--immune regulation in a dynamic and unpredictable antigenic environment. *Annual Review of Immunology* 17:221-253.
37. Van Parijs, L., and A.K. Abbas. 1998. Homeostasis and self-tolerance in the immune system: turning lymphocytes off. *Science* 280:243-248.
38. Van Parijs, L., Y. Refaeli, J.D. Lord, B.H. Nelson, A.K. Abbas, and D. Baltimore. 1999. Uncoupling IL-2 signals that regulate T cell proliferation, survival, and Fas-mediated activation-induced cell death. *Immunity* 11:281-288.
39. Dudley, M.E. 2001. Adoptive transfer of cloned melanoma-reactive T lymphocytes for the treatment of patients with metastatic melanoma. *Journal of Immunotherapy* 24:363-373.
40. Dudley, M.E., J.R. Wunderlich, J.C. Yang, P. Hwu, D.J. Schwartzentruber, S.L. Topalian, R.M. Sherry, F.M. Marincola, S.F. Leitman, C.A. Seipp, L. Rogers-

Freezer, K.E. Morton, A. Nahvi, S.A. Mavroukakis, D.E. White, and S.A. Rosenberg. 2002. A phase I study of nonmyeloablative chemotherapy and adoptive transfer of autologous tumor antigen-specific T lymphocytes in patients with metastatic melanoma. *Journal of Immunotherapy* 25:243-251.

41. Dudley, M.E., J.R. Wunderlich, P.F. Robbins, J.C. Yang, P. Hwu, D.J. Schwartzentruber, S.L. Topalian, R. Sherry, N.P. Restifo, A.M. Hubicki, M.R. Robinson, M. Raffeld, P. Duray, C.A. Seipp, L. Rogers-Freezer, K.E. Morton, S.A. Mavroukakis, D.E. White, and S.A. Rosenberg. 2002. Cancer regression and autoimmunity in patients after clonal repopulation with antitumor lymphocytes. *Science* 298:850-854.
42. Maine, G.N., and J.J. Mule. 2002. Making room for T cells. [letter; comment.]. *Journal of Clinical Investigation* 110:157-159.
43. Hellstrom, K.E., I. Hellstrom, J.A. Kant, and J.D. Tamerius. 1978. Regression and inhibition of sarcoma growth by interference with a radiosensitive T-cell population. *Journal of Experimental Medicine* 148:799-804.
44. Dummer, W., A.G. Niethammer, R. Baccala, B.R. Lawson, N. Wagner, R.A. Reisfeld, and A.N. Theofilopoulos. 2002. T cell homeostatic proliferation elicits effective antitumor autoimmunity. [see comments.]. *Journal of Clinical Investigation* 110:185-192.
45. Tan, J.T., B. Ernst, W.C. Kieper, E. LeRoy, J. Sprent, and C.D. Surh. 2002. Interleukin (IL)-15 and IL-7 jointly regulate homeostatic proliferation of memory phenotype CD8+ cells but are not required for memory phenotype CD4+ cells. [see comments.]. *Journal of Experimental Medicine* 195:1523-1532.
46. Mule, J.J., F.R. Jones, I. Hellstrom, and K.E. Hellstrom. 1979. Selective localization of radiolabeled immune lymphocytes into syngeneic tumors. *Journal of Immunology* 123:600-606.
47. Jameson, S.C. 2002. Maintaining the norm: T-cell homeostasis. *Nature Reviews. Immunology* 2:547-556.
48. Ge, Q., H. Hu, H.N. Eisen, and J. Chen. 2002. Different contributions of thymopoiesis and homeostasis-driven proliferation to the reconstitution of naive and memory T cell compartments. *Proceedings of the National Academy of Sciences of the United States of America* 99:2989-2994.
49. Prlic, M., L. Lefrancois, and S.C. Jameson. 2002. Multiple choices: regulation of memory CD8 T cell generation and homeostasis by interleukin (IL)-7 and IL-15. [letter; comment.]. *Journal of Experimental Medicine* 195:F49-52.
50. Sutmuller, R.P., L.M. van Duivenvoorde, A. van Elsas, T.N. Schumacher, M.E. Wildenberg, J.P. Allison, R.E. Toes, R. Offringa, and C.J. Melief. 2001. Synergism of cytotoxic T lymphocyte-associated antigen 4 blockade and depletion of CD25(+) regulatory T cells in antitumor therapy reveals alternative pathways for suppression of autoreactive cytotoxic T lymphocyte responses. *Journal of Experimental Medicine* 194:823-832.
51. Woo, E.Y., H. Yeh, C.S. Chu, K. Schlienger, R.G. Carroll, J.L. Riley, L.R. Kaiser, and C.H. June. 2002. Cutting edge: Regulatory T cells from lung cancer patients directly inhibit autologous T cell proliferation. *Journal of Immunology* 168:4272-4276.

52. Murakami, M., A. Sakamoto, J. Bender, J. Kappler, and P. Marrack. 2002. CD25+CD4+ T cells contribute to the control of memory CD8+ T cells. *Proc Natl Acad Sci U S A* 99:8832-8837.
53. Liyanage, U.K., T.T. Moore, H.G. Joo, Y. Tanaka, V. Herrmann, G. Doherty, J.A. Drebin, S.M. Strasberg, T.J. Eberlein, P.S. Goedegebuure, and D.C. Linehan. 2002. Prevalence of regulatory T cells is increased in peripheral blood and tumor microenvironment of patients with pancreas or breast adenocarcinoma. *J Immunol* 169:2756-2761.
54. Von Hoff, D.D. 1990. Phase I clinical trials with fludarabine phosphate. *Semin Oncol* 17:33-38.
55. Dimopoulos, M.A., S. O'Brien, H. Kantarjian, S. Pierce, K. Delasalle, B. Barlogie, R. Alexanian, and M.J. Keating. 1993. Fludarabine therapy in Waldenstrom's macroglobulinemia. *American Journal of Medicine* 95:49-52.
56. Feldman, E.J., and M.J. Keating. 1993. Fludarabine in the treatment of lymphoproliferative malignancies. *Cancer Investigation* 11:314-318.
57. Robertson, L.E., and M.J. Keating. 1993. Fludarabine phosphate in the treatment of chronic lymphocytic leukemia: biology, clinical impact, and future directions. *Cancer Treatment & Research* 64:105-119.
58. Plunkett, W., V. Gandhi, P. Huang, L.E. Robertson, L.Y. Yang, V. Gregoire, E. Estey, and M.J. Keating. 1993. Fludarabine: pharmacokinetics, mechanisms of action, and rationales for combination therapies. *Seminars in Oncology* 20:2-12.
59. Keating, M.J., S. O'Brien, L.E. Robertson, H. Kantarjian, M. Dimopoulos, P. McLaughlin, F. Cabanillas, V. Gregoire, L.Y. Yang, and V. Gandhi. 1993. New initiatives with fludarabine monophosphate in hematologic malignancies. *Seminars in Oncology* 20:13-20.
60. Ajani, J.A., J.L. Abbruzzese, J.S. Faintuch, R. Blackburn, B. Levin, and B.M. Boman. 1988. Phase II study of fludarabine phosphate in patients with advanced colorectal carcinoma. *Invest New Drugs* 6:47-50.
61. Kish, J.A., K. Kopecky, M.K. Samson, D.D. Von Hoff, W.S. Fletcher, R.A. Kempf, and F.M. Muggia. 1991. Evaluation of fludarabine phosphate in malignant melanoma. A Southwest Oncology Group study. *Invest New Drugs* 9:105-108.
62. Mittelman, A., R. Ashikari, T. Ahmed, S. Savona, P. Arnold, and Z. Arlin. 1988. Phase II trial of fludarabine phosphate (F-Ara-AMP) in patients with advanced breast cancer. *Cancer Chemother Pharmacol* 22:63-64.
63. Anaissie, E.J., D.P. Kontoyiannis, S. O'Brien, H. Kantarjian, L. Robertson, S. Lerner, and M.J. Keating. 1998. Infections in patients with chronic lymphocytic leukemia treated with fludarabine. *Annals of Internal Medicine* 129:559-566.
64. Cheson, B.D. 1995. Infectious and immunosuppressive complications of purine analog therapy. *Journal of Clinical Oncology* 13:2431-2448.
65. Robertson, L.E., S. O'Brien, H. Kantarjian, C. Koller, M. Beran, M. Andreeff, S. Lerner, W. Plunkett, and M.J. Keating. 1995. A 3-day schedule of fludarabine in previously treated chronic lymphocytic leukemia. *Leukemia* 9:1444-1449.
66. National Cancer Institute, N.I.o.H. 1998. Common Toxicity Criteria V 2.0. http://ctep.info.nih.gov/CTC3/default.htm 2001.

67. Eton, O., S.S. Legha, A.Y. Bedikian, J.J. Lee, A.C. Buzaid, C. Hodges, S.E. Ring, N.E. Papadopoulos, C. Plager, M.J. East, F. Zhan, and R.S. Benjamin. 2002. Sequential biochemotherapy versus chemotherapy for metastatic melanoma: results from a phase III randomized trial. *J Clin Oncol* 20:2045-2052.
68. Creagan, E.T., V.J. Suman, R.J. Dalton, H.C. Pitot, H.J. Long, M.H. Veeder, A.M. Vukov, K.M. Rowland, J.E. Krook, and J.C. Michalak. 1999. Phase III clinical trial of the combination of cisplatin, dacarbazine, and carmustine with or without tamoxifen in patients with advanced malignant melanoma. *J Clin Oncol* 17:1884-1890.
69. Therasse, P., S.G. Arbuck, E.A. Eisenhauer, J. Wanders, R.S. Kaplan, L. Rubinstein, J. Verweij, M. Van Glabbeke, A.T. van Oosterom, M.C. Christian, and S.G. Gwyther. 2000. New guidelines to evaluate the response to treatment in solid tumors. European Organization for Research and Treatment of Cancer, National Cancer Institute of the United States, National Cancer Institute of Canada. *J Natl Cancer Inst* 92:205-216.
