# Supplementary material for: Fludarabine Modulates Immune Response and Extends In Vivo Survival of Adoptively Transferred CD8 T Cells in Patients with Metastatic Melanoma
Source: PLoS One. 2009 Mar 9;4(3):e4749. doi: 10.1371/journal.pone.0004749 (PMC2650617; doi:10.1371/journal.pone.0004749)
Supplement: IRB approval S1 — (0.23 MB PDF) [file pone.0004749.s004.pdf]

PROTOCOL OFFICE
MAILSTOP: LM-230
PHONE: (206) 667-4520

FRED **HUTCHINSON**
CANCER RESEARCH **CENTER**

Institutional Review Board

Continuation Review Report

This shaded box is for IRO only

Institutional Review Board
Fred Hutchinson Cancer Research Center
IRB Committee: ☑ A ☐ B ☐ C
Assurance #: FWA00001920

☐ Full Review ☑ Expedited Review

Review By Date: 12/13/07

Institutional Review Office
Mailstop: J6-110
Telephone: (206) 667-5900

Date Received: IRO REC'D NOV 1[illegible]

Agenda Date: 01/09/08

TITLE OF PROTOCOL: Phase I Study to Evaluate the Safety of Cellular Adoptive Immunotherapy Using Autologous Antigen-Specific T Cell Clones Following Fludarabine Lymphodepletion for Patients with Metastatic Melanoma

IR FILE #:	5596	PROTOCOL # Version date (if applicable)	1796
PRINCIPAL INVESTIGATOR:	Cassian Yee MD	PI MAILSTOP/Phone ext #:	D3-100/667-6287
CONTACT PERSON:	Lisa Schirmer	CONTACT PERSON MAILSTOP/ Phone ext #:	D3-100/667-1539
STUDY DIVISION:	☒ Clinical Research ☐ Basic Sciences ☐ Human Biology	☐ University of Washington Cancer Consortium ☐ Vaccine and Infectious Disease Institute	☐ Public Health Sciences ☐ Other:

CURRENT STATUS OF PROTOCOL (*Check all that apply*):

- ☐ Accrual continuing
- ☐ Accrual complete with treatment intervention, participant interviews, or surveys continuing
- ☐ Research is permanently closed to enrollment of new participants, all participants have completed all research related interventions and research remains active only for long-term follow-up of participants - *Qualifies for Expedited Review**
- ☐ No participants have been enrolled (at any site) and no additional risk(s) have been identified - *Qualifies for Expedited Review**
- ☒ Remaining research activities are limited to data analysis only - *Qualifies for Expedited Review**
- ☐ Closure requested- accrual complete and no further treatment intervention, follow-up, or data analysis required. (Note: When checking this status here, the Investigator submits a Continuation Review Report and includes a final summary under "Findings to Date". No protocol or consent required.
- ☐ Study never activated, closure requested – (Submit this page only with PI Initial/date)
- ☐ Other, Please describe (e.g., previously collected specimens/data/records only study, performing assays):

*Note: Please submit a completed Expedited Review Checklist for Minimal Risk Activities found at http://www.fhcrc.org/intranet/iro/irb/expedited.html if the study's current status qualifies for expedited review".

This shaded box is for IRO only

☑ IRB Approved Documents released date: 11/20/07
☐ IRB Approved Documents not released

[signature] 11/20/07
Signature and Date

JOAN CLARK, M.D., IRB CHAIR, COMM. A
Typed Name and Title

Dates Of Approval 11/20/07 TO ~~11/19/08~~ 11/18/08

VALID ONLY AS LONG AS APPROVED PROCEDURES ARE FOLLOWED

Printed on 11/28/2007
