# Supplementary figures and images for: Fludarabine Modulates Immune Response and Extends In Vivo Survival of Adoptively Transferred CD8 T Cells in Patients with Metastatic Melanoma
Source: PLoS One. 2009 Mar 9;4(3):e4749. doi: 10.1371/journal.pone.0004749 (PMC2650617; doi:10.1371/journal.pone.0004749)

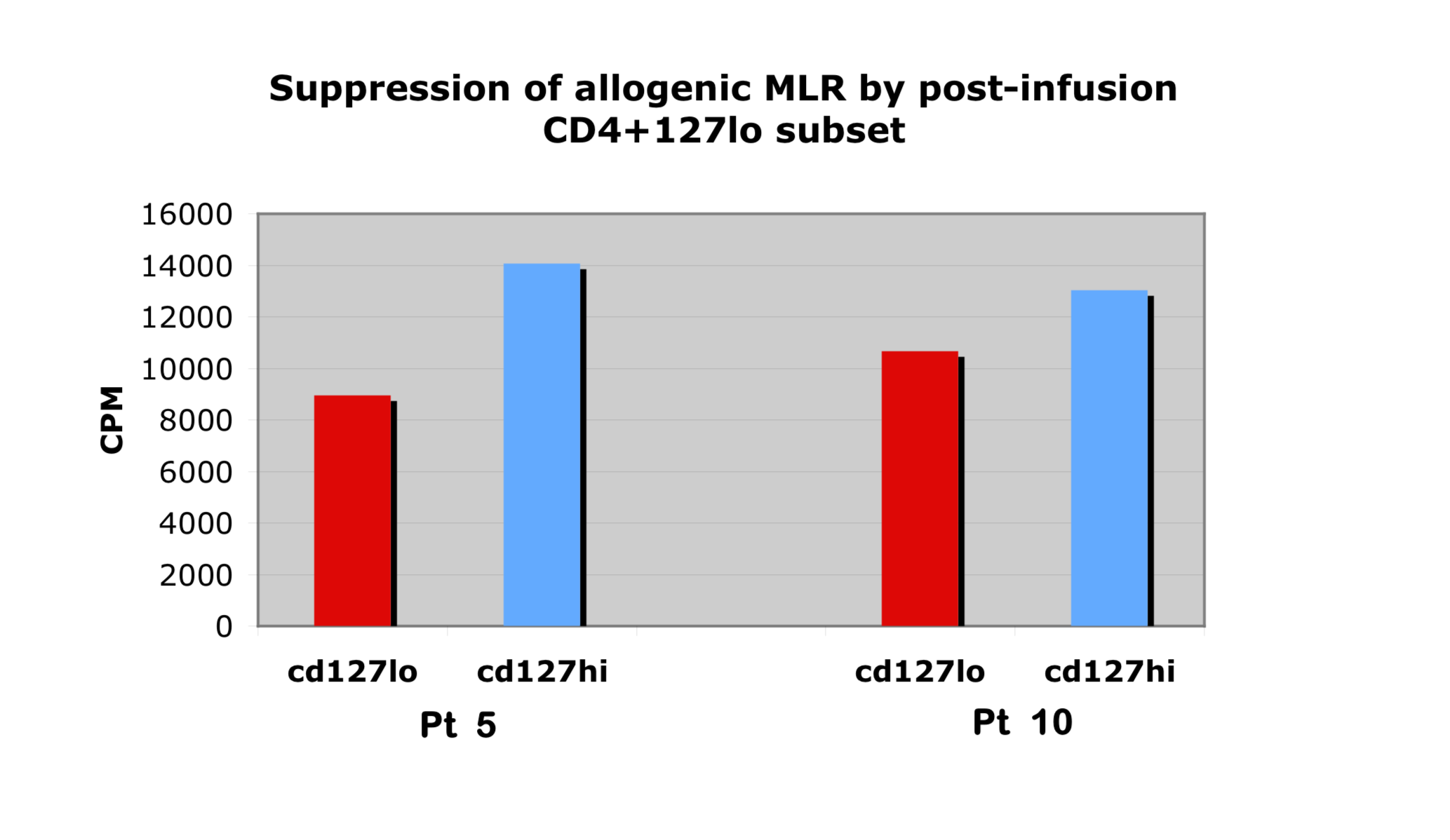

Supplement: Figure S1 — Fludarabine induced regulatory T cells have suppressive capability. Functional analysis from post-infusion PBMC samples of flow cytometry sorted CD4+CD127lo cells. CD4+CD127lo and CD4+CD127hi cells were sorted by flow cytometry in two patients 14 days after T cell infusion. We compared CD127lo and CD127hi subsets in a mixed lymphocyte reaction with autologous responder and irradiated allogeneic stimulator cells. CD127low cells suppress thymidine incorporation in a proliferation assay by 36% in one patient and 18% in another (sorted to responder ratio of 0.3∶1, tested in duplicate). (0.23 MB TIF) [file pone.0004749.s005.tif]
